# Spatiotemporal modelling of hormonal crosstalk explains the level and patterning of hormones and gene expression in *Arabidopsis thaliana* wild-type and mutant roots

**DOI:** 10.1111/nph.13421

**Published:** 2015-04-23

**Authors:** Simon Moore, Xiaoxian Zhang, Anna Mudge, James H Rowe, Jennifer F Topping, Junli Liu, Keith Lindsey

**Affiliations:** 1 The Integrative Cell Biology Laboratory, School of Biological and Biomedical Sciences, Durham UniversitySouth Road, Durham, DH1 3LE, UK; 2 School of Engineering, The University of LiverpoolBrownlow Street, Liverpool, L69 3GQ, UK

**Keywords:** hormonal crosstalk, mathematical modelling, mutant roots, patterning of auxin, PIN proteins, PLS peptide, root development

## Abstract

• Patterning in *Arabidopsis* root development is coordinated via a localized auxin concentration maximum in the root tip, requiring the regulated expression of specific genes. However, little is known about how hormone and gene expression patterning is generated.

• Using a variety of experimental data, we develop a spatiotemporal hormonal crosstalk model that describes the integrated action of auxin, ethylene and cytokinin signalling, the POLARIS protein, and the functions of PIN and AUX1 auxin transporters. We also conduct novel experiments to confirm our modelling predictions.

• The model reproduces auxin patterning and trends in wild-type and mutants; reveals that coordinated PIN and AUX1 activities are required to generate correct auxin patterning; correctly predicts shoot to root auxin flux, auxin patterning in the *aux1* mutant, the amounts of cytokinin, ethylene and PIN protein, and PIN protein patterning in wild-type and mutant roots. Modelling analysis further reveals how PIN protein patterning is related to the POLARIS protein through ethylene signalling. Modelling prediction of the patterning of *POLARIS* expression is confirmed experimentally.

• Our combined modelling and experimental analysis reveals that a hormonal crosstalk network regulates the emergence of patterns and levels of hormones and gene expression in wild-type and mutants.

## Introduction

*Arabidopsis* root development and response to varying environmental conditions involves a complex network of overlapping interactions between plant signalling hormones and gene expression known as ‘hormonal crosstalk’. Hormone concentrations in the cells are a function of multiple factors such as hormone biosynthesis, long- and short-range transport, rate of influx and efflux by carrier proteins, and hormone activation, inactivation and degradation (e.g. Weyers & Paeterson, 2001; Del Bianco *et al*., 2013). Hormones and the associated regulatory and target genes form a network, in which relevant genes regulate hormone activities and hormones regulate gene expression (Chandler, 2009; Depuydt & Hardtke, 2011; Vanstraelen & Benkova, 2012; Bargmann *et al*., 2013). For example, auxin biosynthesis is stimulated by ethylene and inhibited by cytokinins (Eklof *et al*., 1997; Nordstrom *et al*., 2004; Ruzicka *et al*., 2007; Stepanova *et al*., 2007; Swarup *et al*., 2007) and *PIN1* and *PIN2* mRNA and protein concentrations are promoted by auxin and ethylene (Paciorek *et al*., 2005; Vanneste & Friml, 2009) and inhibited by cytokinin (Ruzicka *et al*., 2009). Therefore, root development is controlled by a hormonal crosstalk network that integrates gene expression, signal transduction and the metabolic conversion complexities associated with hormonal crosstalk activity (Liu *et al*., 2014).

Hormone signalling and gene expression responses are patterned to regulate correct root development. Cellular patterning in the *Arabidopsis* root is coordinated in part via a localized auxin concentration maximum close to the quiescent centre (QC; Sabatini *et al*., 1999), which regulates the expression of specific genes such as the *PLETHORA* family (Aida *et al*., 2004) and *WOX5* (Sarkar *et al*., 2007). This auxin gradient has been hypothesized to be sink-driven (Friml *et al*., 2002), and computational modelling suggests that auxin efflux carrier permeability may be sufficient to generate the gradient in the absence of auxin biosynthesis in the root (Grieneisen *et al*., 2007; Wabnik *et al*., 2010; Clark *et al*., 2014). Genetic studies show that auxin biosynthesis (Ikeda *et al*., 2009; Zhao, 2010; Tivendale *et al*., 2014), the AUX1/LAX influx carriers (Swarup *et al*., 2005, 2008; Jones *et al*., 2008; Krupinski & Jonsson, 2010; Band *et al*., 2014) and the PIN auxin efflux carriers (Petrásek *et al*., 2006; Grieneisen *et al*., 2007; Krupinski & Jonsson, 2010; Mironova *et al*., 2010) all play important roles in the formation of auxin gradients. Recently, it has also been demonstrated that growth and patterning during vascular tissue formation in *Arabidopsis* result from an integrated genetic network controlling tissue development (De Rybel *et al*., 2014).

Auxin concentration is regulated by diverse interacting hormones and gene expression and therefore cannot change independently of the various crosstalk components in space and time; similarly, ethylene and cytokinin concentrations and expression of the associated regulatory and target genes are also interlinked (e.g. To *et al*., 2004; Shi *et al*., 2012). Important questions to address in order to understand hormonal crosstalk in root development include how hormone concentrations and expression of the associated regulatory and target genes are mutually related, and how patterning of both hormones and gene expression emerges under the action of hormonal crosstalk. We previously developed a hormonal interaction network for a single *Arabidopsis* cell by iteratively combining modelling with experimental analysis (Liu *et al*., 2010, 2013). We described how such a network regulates auxin concentration in the *Arabidopsis* root by controlling the relative contribution of auxin influx, biosynthesis and efflux, and by integrating auxin, ethylene and cytokinin signalling as well as PIN and POLARIS (PLS) peptide function. The *PLS* gene of *Arabidopsis* transcribes a short mRNA encoding a 36-amino-acid peptide that is required for correct root growth and vascular development (Casson *et al*., 2002). Experimental evidence shows that there is a link between PLS, ethylene signalling, auxin homeostasis and microtubule cytoskeleton dynamics (Chilley *et al*., 2006). *pls* mutant roots are short, with reduced cell elongation, and they are hyperresponsive to exogenous cytokinins. Expression of the *PLS* gene of *Arabidopsis* is repressed by ethylene and induced by auxin, and influences PIN protein abundance in roots (Casson *et al*., 2002; Chilley *et al*., 2006; Liu *et al*., 2013). These and other experimental data reveal that interactions between PLS and PIN are important for the crosstalk between auxin, ethylene and cytokinin (Liu *et al*., 2013).

Mathematical modelling of auxin transport and patterning by constructing multicellular systems in two dimensions previously suggested that correct PIN protein placement is necessary to establish correct auxin patterning (Grieneisen *et al*., 2007; Mironova *et al*., 2012). Here we develop a spatiotemporal model of hormonal crosstalk for the *Arabidopsis* root and show that the level and patterning of auxin, PIN localization and *PLS* gene expression in *Arabidopsis* wild-type and mutant roots can be elucidated by the action of spatiotemporal dynamics of hormonal crosstalk, involving the integration of auxin, ethylene and cytokinin signalling and the functioning of the auxin transporters AUX1 and PIN.

## Materials and Methods

### Plant materials

Wild-type (Col-0, C24) ecotypes and the *pls* and *pls etr1* mutants of *Arabidopsis thaliana* (L.) Heynh have been described previously (Topping & Lindsey, 1997; Casson *et al*., 2002; Chilley *et al*., 2006). *pls* DR5::GFP seedlings were generated by crossing (Liu *et al*., 2010). For *in vitro* growth studies, seeds were stratified, surface-sterilized and plated on growth medium (half-strength Murashige and Skoog medium (Sigma, Poole, UK), 1% sucrose, and 2.5% Phytagel (Sigma)) at 22 ± 2°C as previously described (Casson *et al*., 2009).

### Microscopy and image analysis

Confocal images (for GFP imaging) were taken with a Leica SP5 microscope (Leica Microsystems, Milton Keynes, UK) after counterstaining tissues with 10 mg ml^−1^ propidium iodide as previously described (Casson *et al*., 2009). For image analysis, the mean GUS staining or fluorescence intensity was measured with ImageJ (National Institutes of Health, http://rsb.info.nih.gov/ij). Statistics were carried out using Excel (Microsoft). Results were visualized as average intensities with error bars representing SD of the mean.

### Numerical methods

The set of partial differential equations, which describes spatiotemporal dynamics of hormonal crosstalk in the root (Figs2), is solved using the finite volume method, in which each grid point is used as an element to establish the discrete mass balance equations. The nonlinearity of the reactive terms for all species in the discrete equations is solved by the Picard iteration, and the resulting linear system equations are solved by the preconditioned conjugate-gradient iterative method. The numerical simulations involve two iterations: one for solving the nonlinearity and the other for solving the linear system of equations. In this work, the convergence tolerance for the iteration of solving nonlinearity and for that of solving the linear systems is 10^−5^ and 10^−10^, respectively. Much smaller convergence tolerances for both iterations are also tested, and the numerical results show that further reduction of convergence tolerances for both iterations does not improve the accuracy of numerical simulations.

**Fig. 1:**
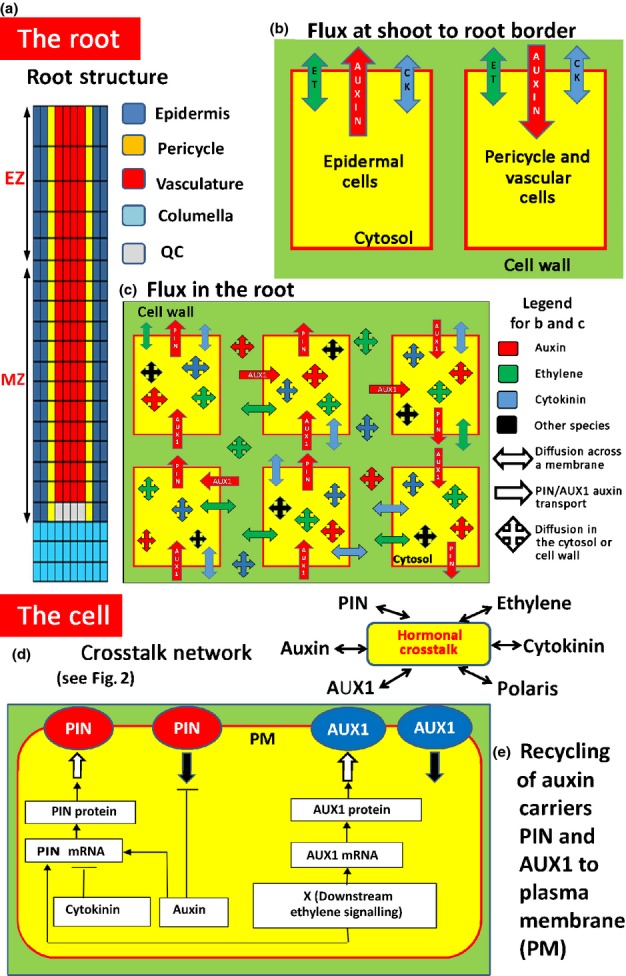
A schematic description of the model that describes *Arabidopsis thaliana* two-dimensional root structure, cell–cell communication and the hormonal crosstalk network in each cell. (a) Multicellular root structure (redrawn and modified from Grieneisen *et al*., 2007) defined by a matrix of grid points (GPs) that form the root map. MZ, meristematic zone; EZ, elongation zone; QC, quiescent centre. (b) Auxin flux by permeability from shoot to root in the pericycle and vascular cell files and from root to shoot in the epidermal files. Ethylene (ET) and cytokinin (CK) flux by diffusion between shoot and root. (c) Species flux between nearest neighbour GP by diffusion within the cytosol (all species) or cell wall (hormones) and hormone flux across the plasma membrane by diffusion (ET and CK) and permeability (auxin). (d) The hormonal crosstalk network in each cell (Fig.2). (e) Dynamic recycling of the auxin carriers PIN and AUX1 by exocytosis and endocytosis to and from the plasma membrane. Auxin inhibits endocytosis of the PIN proteins (Paciorek *et al*., 2005).

**Fig. 2:**
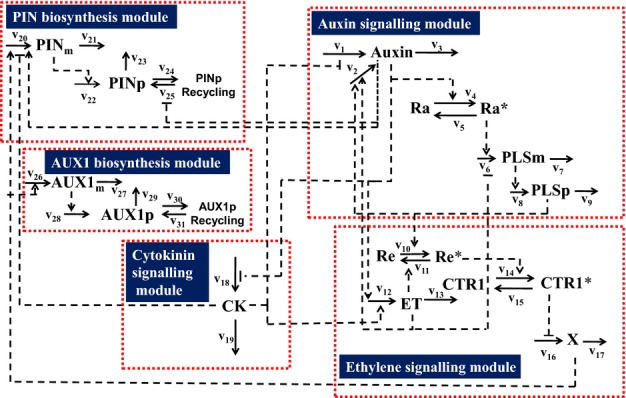
The hormonal crosstalk network in each cell. The network is constructed by adding the AUX1 biosynthesis module to the hormonal crosstalk network we previously developed (Liu *et al*., 2010, 2013). Auxin, auxin hormone; ET, ethylene; CK, cytokinin; PINm, PIN mRNA; PINp, PIN protein; PLSm, POLARIS mRNA; PLSp, POLARIS protein; X, downstream ethylene signalling; Ra*, active form of auxin receptor; Ra, inactive form of auxin receptor; Re*, active form of ethylene receptor, ETR1. Re, inactive form of ethylene receptor, ETR1; CTR1*, active form of CTR1; CTR1, inactive form of CTR1; AUX1 m, AUX1 mRNA; AUX1 p, AUX1 protein.

### Comparison of experimental data and modelling results

In this work, experimental images were analysed using ImageJ (http://imagej.nih.gov/ij). The output of ImageJ is the intensity of each pixel in an experimental image. The relative intensity over the whole image shows the relative hormone response or protein concentration patterning for any measured component. The detailed method for using ImageJ to analyse experimental images is described in Supporting Information Methods S1.

In order to implement the numerical simulations, the root (Fig.1a) was discretized into 2 μm × 2 μm areas, each of which is represented by a grid point. The discrete mass balance equations at each grid point were established, and the spatiotemporal dynamics of all components (hormones, proteins and mRNAs) were analysed (the method for discretizing the root and for implementing numerical simulations is detailed in Methods S2). The outputs of modelling analysis include the concentrations of all components at each grid point and all reaction rates and transport fluxes. Using the concentration of a component (e.g. auxin) at each model grid point, we first calculated the model average of the component over the area described by the root structure (Fig.1a), which was compared with the experimentally measured value of this component. For example, the average model concentration of auxin in the root was compared with the experimentally determined concentration of auxin. Second, we modelled the concentration patterning of the component, represented by a colour map that shows the concentration at each grid point. We compared this result with the experimental image. As an experimental image can represent response rather than concentration itself, we noted this difference when making the comparison. The modelling colour map was directly compared with an experimental image, showing the similarities or differences in patterning. For example, an auxin maximum at or close to the QC in a modelling colour map can be compared with an auxin IAA2::GUS response maximum at or close to the QC. If the maximum in modelling output does not emerge or emerges at a different area, the patterning difference between experimental data and modelling results can be identified. Third, we modelled the concentration profile of the component. In principle, as the concentration at each individual grid point can be calculated from the model, the concentration average can be calculated for any number of grid points. A useful concentration profile can be generated by calculating a series of cross-sectional averages and using them to plot a concentration profile along the longitudinal root axis. This concentration profile can also be generated for different cell types. Similar relative response or concentration profiles can be generated from experimental images using ImageJ. Therefore, we can compare a modelling concentration profile with an experimental response or concentration profile.

To compare a component between wild-type and mutant roots, we focus on trend changes in concentrations, patterning and profile. For example, experimental data show that the auxin concentration in *pls* is lower than that in the wild-type (Chilley *et al*., 2006), and the auxin concentration in *pls etr1* is higher than that in *pls* but lower than that in the wild-type (Chilley *et al*., 2006). As long as modelling results generate the same trend as experimental observations, we considered the modelling result to be similar to or in agreement with experimental measurements.

## Results

### A spatiotemporal model of hormonal crosstalk for *Arabidopsis* root development

Figs1 and 2 schematically describe a multicellular hormonal crosstalk model. The model includes a multicellular root structure (Fig.1a); communication between the multiple root cells (Fig.1b,c); hormonal crosstalk in each cell (Figs1d, 2); and dynamic recycling of the auxin carriers PIN and AUX1 to and from the plasma membrane (Fig.1e). For simplicity, we do not distinguish between the cell wall and the plasma membrane in this work and individual plasma membrane properties are included in cell wall properties. The equations and parameters used to describe the processes in Figs1 and 2 are included in Table S1.

We set up a two-dimensional multicellular root structure using previous work as a starting point (Grieneisen *et al*., 2007). As the lengths of cells in the elongation zone increase proximally (i.e. shootwards from the root meristem; Beemster & Baskin, 1998), we have adapted the root structure previously modelled by Grieneisen *et al*. (2007) to include this feature to describe cell shapes in the *Arabidopsis* root more realistically (Fig.1a). The root structure is defined by a matrix of grid points, each of which has specific properties that define the cytosol or cell wall. Communication between the multiple cells describes how three hormones (auxin, ethylene and cytokinin) and the products of the associated gene expression move in the cytosol, between the cytosol and cell wall, in the cell wall and at the shoot–root boundary (Fig.1b,c; Methods S2). Following previous work (Grieneisen *et al*., 2007; Mironova *et al*., 2012), we consider that auxin is moved out of the cell by the PIN transporter system and into the cell by the AUX1 transporter (Methods S2). Moreover, ethylene and cytokinin diffuse freely across the plasma membrane. All other species are assumed to diffuse only within cytosolic space and cannot diffuse across the plasma membrane into the cell wall. At the shoot–root boundary, following previous work (Grieneisen *et al*., 2007), auxin influx from shoot to root occurs only in the pericycle and vascular cell files. This influx into the root is inhibited by downstream ethylene signalling (designated X in our model), based on experimental evidence, which indicates that a relatively high ethylene signalling response inhibits the transport of auxin from the shoot to the root tip (Suttle, 1988; Chilley *et al*., 2006) (Methods S2). In addition, auxin efflux from the root towards the shoot occurs only in the epidermal cells (Fig.1a). This efflux is facilitated by PIN proteins (Methods S2).

Hormonal crosstalk in the cytosol of each cell describes the production and decay of auxin, ethylene and cytokinin and the products of associated gene expression (mRNA and protein; Figs1d, 2). The regulatory relationships in Figs1(d) and 2 were previously established by iteratively combining experimental measurements with modelling analysis (Liu *et al*., 2010, 2013). In this work, we further consider that AUX1 activities are positively regulated by the downstream ethylene signalling based on experimental observation (7B in Ruzicka *et al*., 2007).

Fig.1(e) describes the dynamic recycling of PIN and AUX1 protein between the cytosol and plasma membrane. Experimental evidence shows that PIN endocytic internalization is inhibited by auxin (Paciorek *et al*., 2005), and so the model includes auxin inhibition of PIN cycling from the plasma membrane to the cytosol.

### Model fitting reveals that both PIN and AUX1 activities must be restricted to certain ranges in order to generate correct auxin patterning

**Fig. 3:**
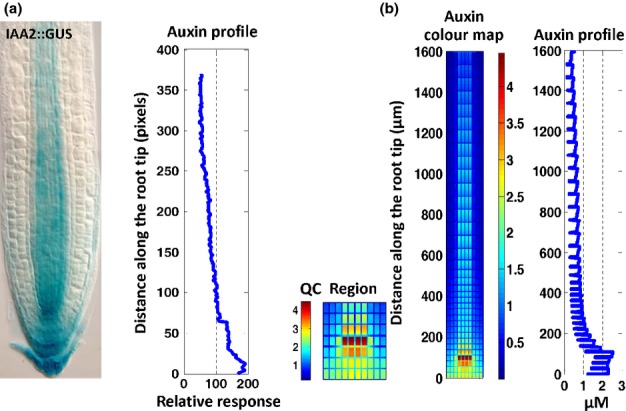
Auxin concentration patterning in the wild-type *Arabidopsis thaliana* root is similar to experimental observation. (a) Experimental image (from Grieneisen *et al*., 2007, with permission) and response profile analysed using Image J. (b) Model concentration colour map and profile (colour bar units, μM). QC, quiescent centre.

The parameters used in this work are included in Table S1. We have used parameter values available in the literature. For example, the diffusion coefficient for auxin is set to 220 μm^2^ s^−1^ (Rutschow *et al*., 2011), the PIN efflux permeability is set to 0.5–5 μm s^−1^, with a median value of 2 μm s^−1^ (Kramer *et al*., 2011) and the AUX1 influx permeability is set to 1.5 ± 0.3 μm s^−1^ (Rutschow *et al*., 2014). It has also been suggested that AUX1 influx must be equal to or greater than PIN efflux, otherwise cells would be depleted of auxin (Kramer, 2004). We chose values for these parameters from these experimental measurements. Parameters relating to ethylene receptor function and CTR1 were studied by Diaz & Alvarez-Buylla (2006), and we used the parameter rate values from their work. Unknown parameter values were adjusted to produce simulation results consistent with experimental data and images and to meet the following criteria: endogenous average auxin concentration for the wild-type root is similar to experimental data; the trend changes in average auxin concentration in wild-type, the *pls* mutant, the *pls etr1* double mutant, and PLS-overexpressing transgenics (PLSox) follow experimental trends (Fig. S1); auxin concentration patterning in the wild-type root is similar to experimental response patterning (Fig.3); the auxin carrier proteins PIN and AUX1 localize predominantly to the plasma membrane (Fig. S2); and cytokinin concentration in the vascular and pericycle cells is higher than that in the epidermal cells (Fig. S3).

Model fitting by manually adjusting unknown parameters reveals that both PIN and AUX1 permeabilities must be restricted to certain ranges in order to generate the auxin concentration patterning that is similar to experimental IAA2::GUS response patterning. For example, if both PIN and AUX1 permeabilities are low, the auxin gradient towards the distal region of the root is gradually smoothed out. If PIN permeability increases, an increase in AUX1 permeability is required to maintain a similar auxin patterning to that in experimental data (Fig. S4). Although the auxin gradient has been hypothesized to be sink-driven (Friml *et al*., 2002) and computational modelling suggests that auxin efflux carrier permeability may be sufficient to generate the gradient (Grieneisen *et al*., 2007; Wabnik *et al*., 2010), recent work shows that AUX1 is also essential to create the auxin gradient at the root tip (Band *et al*., 2014). Our modelling results support the view that both PIN and AUX1 permeabilities work together to generate auxin patterning. If AUX1 permeability is not varied in the model such that it becomes a limiting factor for auxin transport, the importance of AUX1 permeability for generating an auxin gradient cannot be revealed. In a previous study, effects of varying AUX1 permeability were not reported (Grieneisen *et al*., 2007).

Model fitting also reveals that, if cytokinin is allowed to be synthesized in all cells, cytokinin concentration in the epidermal cells is higher than in the vascular and pericycle cells. If we consider that ARR5::GUS signalling reflects cytokinin concentration, then the modelling result is different from experimental measurement. However, if cytokinin biosynthesis occurs predominantly in the vascular and pericycle cells (modelled by limiting synthesis to the vascular and pericycle cells), the modelled cytokinin concentration in the vascular and pericycle cells is higher than that in the epidermal cells, a result similar to experimental observations. Nevertheless, the trend of cytokinin patterning along the longitudinal root axis still differs between the experimental images and modelling results (Fig. S3). These results may indicate that cytokinin biosynthesis is predominantly restricted to the vascular and pericycle cells, by an as yet poorly understood regulatory mechanism, which is supported by experimental evidence indicating that cytokinin biosynthesis may be tissue-specific (Miyawaki *et al*., 2004). The difference in longitudinal cytokinin patterning suggests possible additional unknown regulatory factors that influence patterning along the root axis. In this work, we allow cytokinin biosynthesis to occur only in the vascular and pericycle cells.

An example of model-fitting outcomes is shown in Fig.3 and all other model-fitting results are included in Fig. S2–S4. Fig.3 shows that the modelled auxin concentration patterning in the wild-type *Arabidopsis* root is similar to the experimentally determined auxin IAA2::GUS response patterning, with an auxin maximum established at or close to the QC (Grieneisen *et al*., 2007). The modelled auxin concentration profile is also similar to the auxin IAA2::GUS response profile generated from the Grieneisen *et al*. (2007) experimental image. Moreover, we have analysed auxin concentration profiles for each of the three different types of cell in the model (epidermal, pericycle and vascular) shown in Fig.1(a). Fig. S5 shows that the concentration profiles for the three cell types follow similar trends to that in Fig.3. Moreover, an auxin maximum is predominantly established in the central tissues at or close to the QC.

Experiments have shown that the auxin response can be regulated by different effectors, and therefore is not necessarily equivalent to auxin concentration (Vernoux *et al*., 2011; Cho *et al*., 2014). Although our modelling results (Figs3, S5) are similar to auxin IAA2::GUS response, we further experimentally measured auxin DII-VENUS response (Fig. S6) and compared it with our modelling results. Fig. S6 shows that in the meristematic zone and QC, the modelled concentration profile is similar to the experimental auxin response profile derived from the DII-VENUS response. However, in the elongation zone, the modelled concentration profile is not in agreement with experimental DII-VENUS imaging. In Notes S1, we further discuss the comparison between modelling results and experimental DII-VENUS response. In particular, we compare our modelling results with experimental observations in the literature (Brunoud *et al*., 2012; Band *et al*., 2014) (Notes S1). Our analysis shows that our modelling results are in reasonably good agreement with experimental data. Specifically, the trend of the modelled auxin concentrations for five cell types (i.e. QC, stele, endodermis, epidermis meristem and cortex meristem) is similar to the trend observed experimentally (1K in Band *et al*., 2014). Modelling also shows that the vascular, pericycle and epidermal cells have high, medium and low relative auxin concentrations, respectively (Notes S1). This trend is in agreement with experimental observations (2B in Brunoud *et al*., 2012). In Notes S1, we also discuss the discrepancies between our modelling results and the experimental observations of DII-VENUS response.

Therefore, auxin concentration patterning generated by our model, with an auxin maximum established at or close to the QC, is similar to both experimental IAA2::GUS and DII-VENUS response patterns (Figs3, S6).

After the model was parameterized following these model-fitting criteria, a wild-type root was defined. We further evaluated model sensitivity (Notes S2), showing that modelling results are robust to variations in parameter values. We then used the model to study the level and patterning of hormones and gene expression in *Arabidopsis* roots.

### Auxin flux from shoot to root and auxin patterning in the *aux1* mutant

**Fig. 4:**
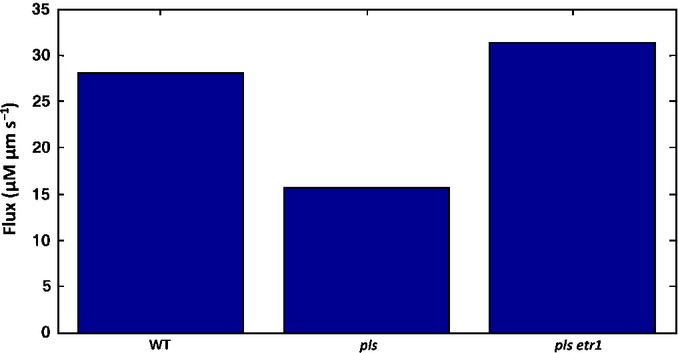
Modelling prediction of auxin flux from the shoot to the root is similar to experimental measurements for *Arabidopsis thaliana* (4e in Chilley *et al*., 2006. WT, wild-type. http://www.plantcell.org, Copyright American Society of Plant Biologists).

Assuming that rootward auxin flux measured in inflorescence stem segments is similar to shoot-to-root auxin flux at the shoot–root boundary, experimental measurements of the shoot-to-root auxin flux in inflorescence stem segments for wild-type, *pls* mutant and *pls etr1* double mutant show that auxin flux from shoot to root in the *pls* mutant is significantly lower than that for wild-type. This effect reduces the total amount of auxin in the root tip and reduces auxin responses in the *pls* root. The auxin flux into the root, and the root auxin content for the *pls etr1* double mutant also recover approximately to the value of the wild-type (4e in Chilley *et al*., 2006). Although the modelled auxin flux into the root for the *pls etr1* double mutant is slightly higher than that for the wild-type, our modelling analysis exhibits a similar trend to experimental observation (Fig.4). Therefore, spatiotemporal dynamics of hormonal crosstalk correctly predicts shoot-to-root auxin flux in different genotypes.

**Fig. 5:**
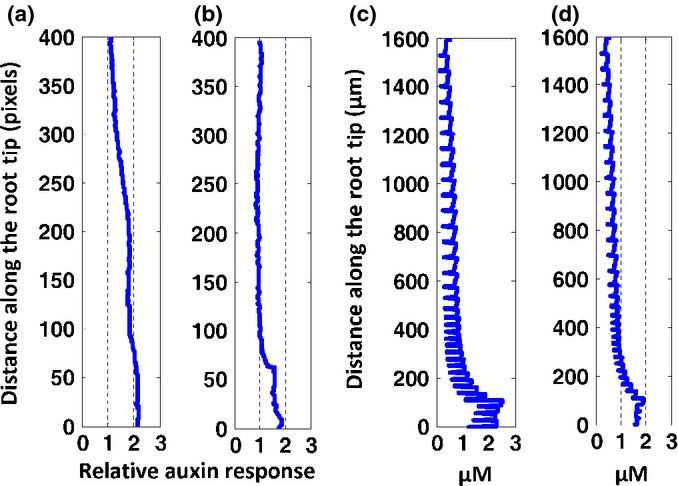
Spatiotemporal modelling of hormonal crosstalk correctly predicts auxin patterning in the *aux1* mutant of *Arabidopsis thaliana*. (a, b) Auxin response profiles for the wild-type (a) and the *aux1* mutant (b). We calculated response profiles using experimental images (2 in Swarup *et al*., 2001). (c, d) The corresponding modelling results of auxin concentration profiles for the wild-type (c) and the *aux1* mutant (d).

Analysis of the experimental images of auxin response patterning in the wild-type and the *aux1* mutant shows a decrease in auxin response in the root for *aux1* compared with the wild-type (Fig.5a,b), consistent with experimental auxin assays (Swarup *et al*., 2001). By considering that in addition to AUX1, there are other auxin influx carriers (such as LAX proteins) which are not described in the model, we assume that auxin influx permeability in the *aux1* mutant is reduced by 50%. Auxin concentration profiles generated by modelling (Fig.5c,d) are similar to the corresponding experimental auxin response profiles (Fig.5a,b). In both modelled and experimental profiles, the auxin concentration or response maximum for *aux1* is slightly lower than that for the wild-type in the QC region, and in both *aux1* and the wild-type, auxin concentrations or responses decrease towards the proximal region of the root tip to reach approximately the same value. Therefore, the model for spatiotemporal dynamics of hormonal crosstalk correctly predicts auxin patterning in the *aux1* mutant. This indicates that integration of auxin influx permeability into the hormonal crosstalk is able to explain auxin patterning in specific mutants.

### Concentrations of cytokinin, ethylene and PIN protein

**Fig. 6:**
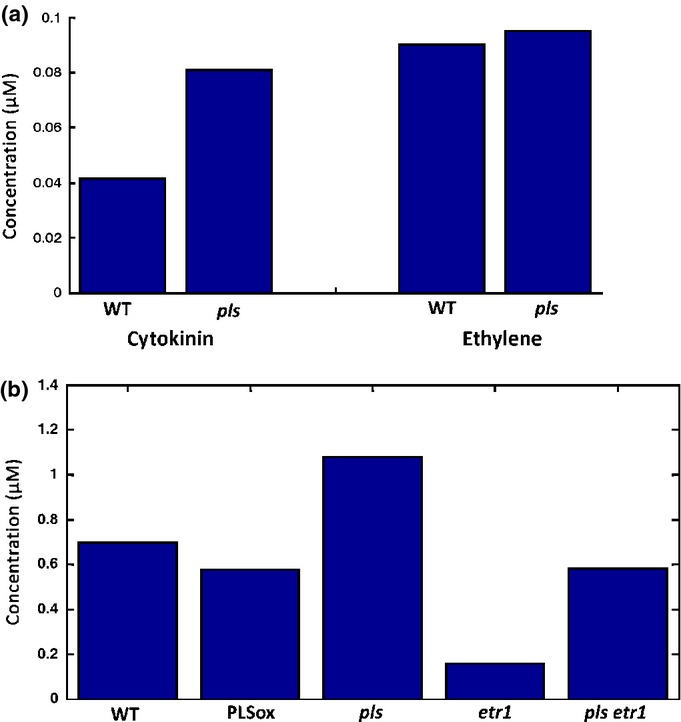
Modelling predictions of the average concentrations of cytokinin and ethylene hormones and the PLS protein in *Arabidopsis thaliana*. (a) Modelling predictions of the average concentrations of cytokinin and ethylene in the *pls* mutant. (b) Modelling predictions of the average concentrations of PIN protein in PLSox transgenics, *pls*, *etr1* mutants and the *pls etr1* double mutant.

Auxin can negatively regulate cytokinin biosynthesis (Nordstrom *et al*., 2004). The accumulated concentration of cytokinin is described in the hormonal crosstalk network as the balance between its biosynthesis and its removal (Fig.2). Fig.6(a) predicts that, in the *pls* mutant, the average endogenous cytokinin concentration for the root is increased to *c*. 1.9-fold of that in wild-type. Experimental measurements show that different cytokinins have significantly different fold changes. However, the general trend is that endogenous cytokinin concentrations in the *pls* mutant are significantly increased, with a median fold change of 1.42 (Table 1 in Liu *et al*., 2010).

Experimentally it has been shown that *PLS* transcription does not affect ethylene concentration (Chilley *et al*., 2006) and this result is in agreement with our simulations (Fig.6a). In addition, the relative PIN protein concentrations in wild-type, PLSox and *pls*, *etr1* and *pls etr1* mutants were experimentally measured (1in Liu *et al*., 2013). The relative average root concentrations predicted by the model show similar trends to those observed experimentally (Fig.6b).

In conclusion, modelling predictions for the average concentrations of cytokinin, ethylene and PIN protein in *Arabidopsis* wild-type and mutant roots are in agreement with experimental observations, suggesting that the concentrations of hormones and proteins are controlled by the integrative system of hormonal crosstalk (Figs2).

### PIN patterning in *Arabidopsis* wild-type and mutant roots

As it was possible to explain the average PIN protein concentration in different mutants using the spatiotemporal model of hormonal crosstalk (Fig.6), we went on to ask whether PIN patterning in the *Arabidopsis* root is also controlled by the integrative system of hormonal crosstalk (Figs2). To address this question, we compared experimental evidence for PIN1 and PIN2 patterning with modelling predictions.

The relative concentration data were extracted from experimental images of PIN1 and PIN2 protein localization in wild-type, *pls, etr1* and PLSox seedlings and the *pls etr1* double mutant (1a in Liu *et al*., 2013). Data were plotted as PIN concentration profiles for comparison with modelling results.

**Fig. 7:**
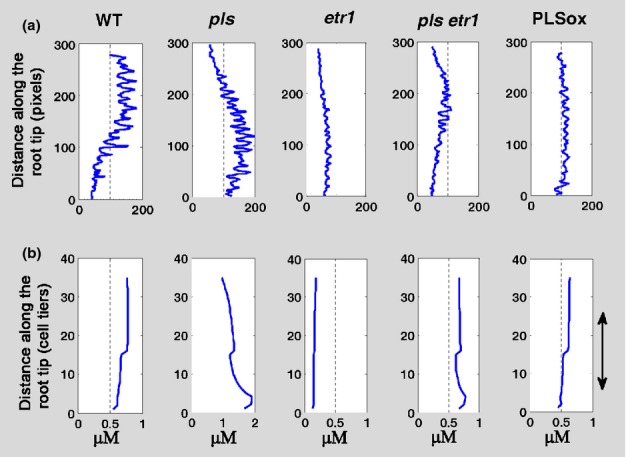
Patterning of PIN1 protein expression in *Arabidopsis thaliana*. (a) Patterning of PIN1 protein by analysing the experimental images (2 in Liu *et al*., 2013). (b) Modelling prediction of the patterning of PIN1 protein. The experimental images (a) represent a region in the root from approximately five to 25 cell tiers from the tip. In (b), this region is denoted by the arrow.

PIN1 protein is localized in the root mainly in the vascular cells (Blilou *et al*., 2005). PIN1 concentration profiles were generated using the experimental data from the vascular tissues only and compared with the corresponding profiles from the vascular and pericycle cells in the model (Fig.1). PIN1 protein predominantly localizes to, and is active in, the plasma membrane. A modelled concentration profile based on each grid point tends to mask trend changes resulting from large variations in PIN1 concentration between the plasma membrane and cytosol. Therefore, to smooth out the concentration differences and more clearly demonstrate PIN1 trends in the model, we calculated the average PIN1 concentration for each cell tier cross-section of the root, rather than for cross-sections at each grid point position along the longitudinal root axis. The experimental images (1a in Liu *et al*., 2013) represent a region of approximately five to 25 cell tiers from the tip. A similar region in modelling outputs is marked by the arrow in Fig.7(b). The trends in Fig.7(a), derived from the experimental images, should be approximately compared with the region marked by the arrow in Fig.7(b). As shown in Fig.7, the trends of PIN1 patterning in experimental images of wild-type and mutant roots were found to be similar to the corresponding outcomes of modelling simulations, suggesting that PIN1 patterning is a result of the action of hormonal crosstalk in wild-type and mutant/PLSox roots.

Modelling analysis further revealed that changes in PIN1 patterning in wild-type and mutant/PLSox roots reflect changes in the *PIN1* transcription rate resulting from different contributions of auxin, ethylene and cytokinin. For example, modelled PIN1 patterning in the wild-type shows that the amount of PIN1 generally decreases from the proximal region to the distal region of the root (Fig.7a). However, in the *pls* mutant, an opposite trend emerges (Fig.7a). Model calculation shows that, in the *pls* mutant, the *PIN1* transcription rate has significantly increased at the region near the root tip (Fig. S7). Further modelling analysis reveals that, in the wild-type, the downstream component of ethylene signalling, designated X, is suppressed as a result of the action of PLS at the region near the tip (Fig. S8). PLS patterning displays an increasing abundance from the proximal to the distal end of the root, predominantly as a result of the regulation of *PLS* expression by auxin (Fig. S8; also see the section ‘Modelling prediction of *POLARIS* expression pattern is confirmed by experiments). In the *pls* mutant, the suppression of X is relaxed owing to the loss of PLS function. This enhances the rate of PIN1 biosynthesis at the region near the tip and therefore PIN1 patterning shows an increasing concentration trend from the proximal to the distal region. In addition, in the *pls* mutant, the auxin concentration decreases (Fig. S1) and the cytokinin concentration increases (Fig.6). As auxin positively regulates, and cytokinin negatively regulates, *PIN1* transcription, the increase in *PIN1* transcription rate at the region near the tip also reflects the effects of both auxin and cytokinin signalling.

Therefore, the overall effects of auxin, ethylene and cytokinin result in opposite trends in PIN1 patterning in wild-type and *pls* mutant roots. This example demonstrates that spatiotemporal hormonal crosstalk, which describes simultaneous actions of multiple hormones and the associated genes, is necessary for specifying the patterning of PIN1 in the root. Fig.7 further shows that the modelled patterning trend of PIN1 for wild-type, *pls*, *etr1* and PLSox (the region is denoted by the arrow) is similar to the corresponding experimental trend. However, a noticeable difference for the *pls etr1* double mutant can be identified. This indicates the limitation of our model for analysing this double mutant.

Patterning of PIN2 protein was also analysed (Fig. S9). Modelling predictions on the patterning of PIN2 protein for the wild-type and PLSox are in reasonable agreement with experimental data. However, discrepancies between modelling results and experimental data emerge for other mutants. In Fig. S9, we describe and discuss these results further.

### Modelling prediction of *POLARIS* expression pattern is confirmed by experiments

As shown in Fig.2, the *PLS* gene of *Arabidopsis*, which transcribes a short mRNA encoding a 36-amino-acid peptide (Casson *et al*., 2002; Chilley *et al*., 2006), is important for establishing crosstalk among auxin, ethylene, and cytokinin. Here we used both experimental analysis and modelling to investigate further the control of the patterning of *PLS* gene expression.

**Fig. 8:**
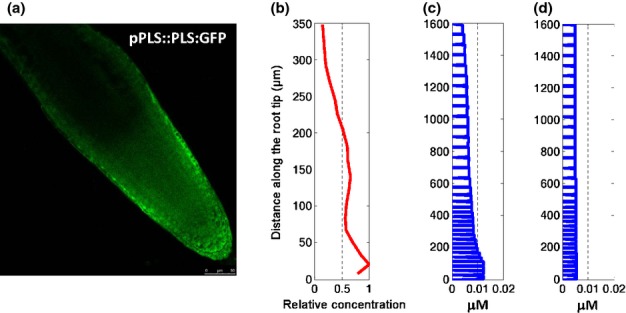
Experimental and modelling results for the patterning of *PLS* gene expression in *Arabidopsis thaliana*. (a) Image of *PLS* gene expression. (b) PLS protein concentration profile. (c) Modelling prediction of the PLS protein profile. (d) Modelling prediction of the PLS protein profile if auxin regulation of *PLS* transcription is removed from the hormonal crosstalk network.

Experimental imaging of PLS protein accumulation in the wild-type root (Fig.8a) shows a concentration maximum near the distal region, with the concentration declining towards the proximal region of the root. This is similar to the expression of the *PLS* gene as monitored by *PLS* promoter-GUS analysis (Casson *et al*., 2002; Chilley *et al*., 2006). The PLS concentration profile generated from the experimental fluorescence image (Fig.8a) illustrates this patterning graphically (Fig.8b). The spatiotemporal modelling of hormonal crosstalk predicts the same trend (Fig.8c), indicating that the hormonal crosstalk network (Fig.2) controls the patterning of *PLS* gene expression and protein accumulation. Modelling calculations reveal that the rate of *PLS* transcription reaches a maximum in the distal part of the root (Fig. S10), resulting in the patterning of *PLS* expression (Fig.8). As indicated in Fig.8(d), if *PLS* transcription is not regulated by auxin, the modelled patterning of *PLS* expression is not in agreement with experimental observation. This reflects the predominant role of auxin in the regulation of *PLS* expression.

### Ethylene and AUX1 patterning in *Arabidopsis* wild-type root

Modelling prediction of the endogenous ethylene concentration patterning is similar to experimentally determined response patterning (Martin-Rejano *et al*., 2011). Both modelling and experimental results show increases in ethylene responses towards the proximal part of the root (Fig. S11).

In this work, we consider that AUX1 activity is positively regulated by the downstream ethylene signalling based on experimental observation (7B in Ruzicka *et al*., 2007). Model results for AUX1 patterning (Fig. S12) are, in part, similar to experimental imaging (Fig. S8 in Band *et al*., 2014) with AUX1 concentrations increasing proximally in the epidermis, and higher AUX1 concentrations in the outer cell layers than in the central cell cylinder. Experimentally, it has been shown that, within the epidermis, AUX1 is present mainly in the elongation zone cells (Band *et al*., 2014). However, the model does not exhibit the elevated experimental AUX1 concentrations in the columella and near the QC or the proximally declining AUX1 concentrations in the central cylinder. The differences between modelling and experimental results might indicate that, in addition to ethylene, other effectors also regulate AUX1 activity.

## Discussion

Experimental information accumulated over many years indicates that, in root development, hormones and the associated regulatory and target genes form a network in which relevant genes regulate hormone activities and hormones regulate gene expression. Functionally important patterns of hormone distribution, hormone responses and gene expression are presumed to emerge from these interactions. However, little is known about how this patterning is generated. By developing an integrative model that combines experimental data, the construction of a hormonal crosstalk network, a spatial root structure for cell–cell interactions and spatiotemporal modelling, we demonstrate that the spatiotemporal dynamics of hormonal crosstalk establishes the causal relationship for the amount of auxin, ethylene, cytokinin, PIN protein and PLS protein, as well as the mechanisms for generating patterning in these hormones and proteins.

In this work, we set up a two-dimensional multicellular root structure using previous work as a starting point (Grieneisen *et al*., 2007). Although the root structure described by Fig.1(a) is a representative description of *Arabidopsis* root, it is incomplete and lacks a lateral root cap. Future research could therefore include additional features to understand, for example, how a lateral root cap contributes to the spatiotemporal dynamics of hormonal crosstalk, and how the spatiotemporal dynamics of hormonal crosstalk is formed in a three-dimensional multicellular root structure with the subcellular resolution. Experiments have shown that a lateral root cap is important for transporting auxin from the apical area to the elongation zone (Swarup *et al*., 2005; Band *et al*., 2014).

**Fig. 9:**
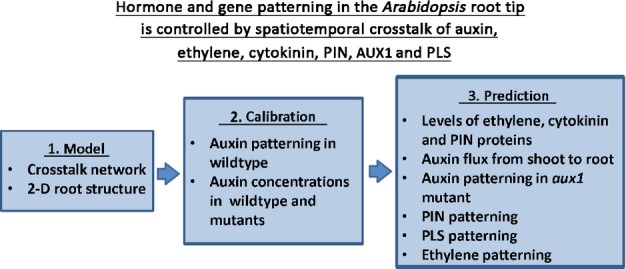
A summary of how spatiotemporal modelling of hormonal crosstalk explains the level and patterning of hormones and gene expression in *Arabidopsis thaliana* wild-type and mutant roots. 2-D, two-dimensional.

The work presented in this paper provides a framework for studying the level and patterning of hormone distribution, hormone responses and gene expression by iteratively combining experimental data with the construction of a hormonal crosstalk network, a spatial root structure for cell–cell interactions and spatiotemporal modelling. We show that the level and patterning of auxin, ethylene and cytokinin responses, and expression of *PIN*s and *PLS* can be explained by spatiotemporal hormonal crosstalk in the *Arabidopsis* root, as summarized in Fig.9.

Experimental analysis has shown that PIN content in *Arabidopsis* varies in response to a range of hormones. Auxin positively regulates amounts of several PIN proteins in different developmental contexts (Blilou *et al*., 2005; Laskowski *et al*., 2006; Chapman & Estelle, 2009; Vanneste & Friml, 2009) by a signalling pathway regulating transcription (Woodward & Bartel, 2005), and also by promoting accumulation at the plasma membrane (Paciorek *et al*., 2005). Ethylene also up-regulates *PIN*s (e.g. *PIN2*, Ruzicka *et al*., 2007) while cytokinin negatively regulates *PIN1*, *PIN2* and *PIN3* (Ruzicka *et al*., 2009; Bishopp *et al*., 2011a), but positively regulates *PIN7*. In this work, we concentrate on the investigation of PIN1 and PIN2. In addition, as PIN3 (which is negatively regulated by cytokinin) and PIN7 (which is positively regulated by cytokinin) are localized at similar positions (Ruzicka *et al*., 2009; Bishopp *et al*., 2011a) in the root, it may be reasonable to assume that the o verall effects of cytokinin on both PIN3 and PIN7 have little net effect on auxin transport. PIN concentrations are also influenced by other genes. For example, in the *pls* mutant, both PIN1 and PIN2 increase (Liu *et al*., 2013). It is also evident that ethylene activates the biosynthesis of auxin locally in the root tip (Stepanova *et al*., 2007; Swarup *et al*., 2007), and that both auxin and cytokinin can synergistically activate the biosynthesis of ethylene (Chilley *et al*., 2006; Stepanova *et al*., 2007). Numerous experimental analyses have shown that auxin patterning, with a localized concentration maximum in the root tip, is pivotal for correct root development (Sabatini *et al*., 1999), and that hormonal interactions determine PIN localization patterns (Liu *et al*., 2013).

During *Arabidopsis* root development, both the amount and patterning of proteins are interlinked. In the wild-type root, PIN1 concentrations generally decrease from the proximal to the distal region (Fig.7) and PLS generally increases from the proximal end to the distal end (Fig.8). However, in the *pls* mutant, PIN1 concentrations generally increase from the proximal end to the distal end. In addition, in the *pls* mutant, the average auxin, ethylene and cytokinin concentration or response in the root is reduced, remains approximately constant, and is increased, respectively (Chilley *et al*., 2006; Liu *et al*., 2010), while the average PIN1 concentration increases (Liu *et al*., 2013). This work shows that the causal relationship between the level and patterning of PIN1 and PLS proteins can be established by studying the spatiotemporal dynamics of hormonal crosstalk.

In order for the root to generate auxin patterning similar to experimental results, the permeability of both the PIN and AUX1 auxin carrier proteins is important and must be limited to certain ranges. It can be concluded that both PIN and AUX1 proteins work together to generate auxin patterning similar to experimental results. It has been suggested that AUX1 influx must be at least equal to PIN efflux to avoid auxin depletion in the cells (Kramer, 2004). Previous modelling results have suggested that either the auxin efflux carrier PIN activity (Grieneisen *et al*., 2007; Wabnik *et al*., 2010) or the AUX1 activity (Band *et al*., 2014) are essential to create the auxin gradient at the root tip. Our results suggest that, owing to the action of a hormonal crosstalk network, the coordination of AUX1 and PIN activity is related to many aspects of PIN and AUX1 proteins, including transcription, translation, decay and recycling of the AUX1 to PIN proteins between the plasma membrane and intracellular compartments.

The discrepancy between experimental and modelling results for cytokinin patterning suggests that there are unknown molecular mechanisms for regulating cytokinin biosynthesis and/or degradation, and further experimental investigations are required to elucidate these mechanisms. The rate-limiting step for cytokinin biosynthesis involves a group of isopentenyltransferase (IPT) enzymes. While *IPT* genes are expressed throughout the root, different genes appear to display tissue-specific expression at different levels. In the root, *IPT* genes are predominantly expressed in the xylem precursor cells, the phloem tissue, the columella and the endodermis of the elongation zone (Miyawaki *et al*., 2004). This expression patterning appears to be supported by ARR5::GUS cytokinin response imaging (Fig. S3). In this image, ARR5::GUS cytokinin response in the epidermal and cortical cells is much lower than that in the central cells. Experimental evidence therefore indicates that cytokinin biosynthesis could be tissue-specific. In our model, cytokinin biosynthesis was restricted to the central pericycle/border, vascular and columella cells.

Our modelling results for cytokinin concentration patterning (Fig. S3) are quantitatively different from experimental observations (revealed as *ARR5::GUS* expression, as a proxy for cytokinin distribution; Werner *et al*., 2003). The modelled cytokinin concentration increases from the distal to the proximal region of the root. This patterning is consistent with the reduction of auxin concentration/response from the distal to the proximal region, as described in our hormonal crosstalk network (Fig.2) where auxin negatively regulates cytokinin biosynthesis based on experimental observations (Nordstrom *et al*., 2004). However, this cytokinin patterning is opposite to the data based on experimental images (Werner *et al*., 2003).

This discrepancy leads to the following possibilities. First, the experimental data (Nordstrom *et al*., 2004) show that the auxin-mediated regulation of cytokinin biosynthesis is different for iP and Z types. While biosynthesis of the Z type is inhibited by auxin, the iP type may not be inhibited by auxin. Thus, a detailed description of the regulatory relationship between auxin concentration and cytokinin biosynthesis for root development requires experimental measurement to determine the location of specific types of cytokinin in the root, and then to derive how cytokinin biosynthesis and degradation are regulated at each location. Second, the cytokinin patterning derived from *ARR5::GUS* images (Werner *et al*., 2003) might not accurately represent the patterning of cytokinin concentration and therefore might not be directly comparable to modelled patterning of cytokinin concentration. The *ARR5::GUS* images measure the activation of the *ARR5* promoter by cytokinin, therefore indicating the activity of cytokinin signalling rather than cytokinin concentration. Bishopp *et al*. (2011a) have discussed that AHP6, which inhibits the cytokinin signalling pathway and *ARR5* expression, is regulated by auxin in the xylem axis. This could indicate that *ARR5::GUS* images represent the effects of both cytokinin and AHP6 concentration, and therefore might not solely reflect cytokinin concentration. Third, it has been demonstrated that cytokinin is transported from the shoot to the root in the phloem (Bishopp *et al*., 2011b), which, in combination with local biosynthesis, degradation and diffusion, could influence cytokinin concentration and signal patterning in the root tip. Interestingly, in a different context for root development analysis, it has also been shown that an additional component is required to position cytokinin signal patterning (Muraro *et al*., 2014). Therefore, the combination of our analysis in this work with the information in the literature indicates that the patterning of cytokinin concentration and signalling requires further experimental and modelling studies.

Based on experimental results (Nordstrom *et al*., 2004), our hormonal crosstalk network (2, Liu *et al*., 2013) describes a negative regulation of auxin biosynthesis by cytokinin. However, Jones *et al*. (2010) have shown that cytokinin positively regulates auxin biosynthesis in young developing tissues (10 d after germination). In previous work, our hormonal crosstalk network analysis revealed that both sets of experimental results (Nordstrom *et al*., 2004; Jones *et al*., 2010) can be incorporated into the hormonal crosstalk network, leading to the same conclusions about other regulatory relationships of hormonal crosstalk (Liu *et al*., 2013). In the current research, we have analysed both cases using the same spatial setting (Fig.1) and our modelling results indicate that each leads to qualitatively similar results. Therefore, the conclusions we have drawn in this work are applicable to both cases. In the current paper, we have concentrated on an analysis based on the experimental results of Nordstrom *et al*. (2004).

In root development, the complexity of hormonal signalling includes many aspects. This work has shown that the spatiotemporal dynamics of hormonal crosstalk, which integrates hormonal crosstalk at a cellular level with root structure, is able to explain two important aspects – the steady-state level and the patterning of hormones/hormone responses and gene expression. Recent studies have shown that growth and hormonal patterning can affect each other (De Rybel *et al*., 2014; Mahonen *et al*., 2014). Future research should investigate the spatiotemporal dynamics of hormonal crosstalk in the presence of, or in response to, growth.

All recent modelling and experimental work (Chickarmane *et al*., 2010; Bargmann *et al*., 2013; Hill *et al*., 2013; De Rybel *et al*., 2014) shows that integration of regulatory networks into spatial root structures is a promising tool for elucidating mechanisms of development. By integrating other genes into the hormonal crosstalk network (Mintz-Oron *et al*., 2012; Bargmann *et al*., 2013; Hill *et al*., 2013; De Rybel *et al*., 2014) and by expanding root structure to include more details of cell-to-cell communication (Chickarmane *et al*., 2010; Hill *et al*., 2013; De Rybel *et al*., 2014), we should in future be able to elucidate the level and patterning of other hormones and gene expression.

## Supporting Information

Fig. S1 Trend in average root auxin concentration in wild-type and mutant plants. **Fig. S2** Modelling results show that PIN and AUX1 auxin carrier proteins localize predominantly to the plasma membrane in the wild-type. **Fig. S3** Cytokinin images and concentration profiles. **Fig. S4** Auxin patterning for different combinations of PIN and AUX1 permeability. **Fig. S5** Modelled auxin concentration profiles for the three different cell types (epidermal, pericycle and vascular cells). **Fig. S6** DII-VENUS response profile measured from the experimental image, compared with the model auxin concentration profile for the wild-type root. **Fig. S7** Modelling results for PINm transcription rates in the wild-type. **Fig. S8** Modelling results for patterning of X, downstream of ethylene signalling, and PLSp, POLARIS protein, in the wild-type. **Fig. S9** Comparison of experimental and modelling PIN2 patterning for wild-type and mutant plants. **Fig. S10** Modelling results for PLSm transcription patterning in the wild-type. **Fig. S11** Modelling prediction of ethylene patterning is similar to experimental measurements. **Fig. S12** Modelled AUX1 concentration profiles for the three different cell types (epidermal, pericycle and vascular cells). **Table S1** Model equations and parameter values for the model described in 1 and 2 **Methods S1** Using ImageJ to analyse experimental images. **Methods S2** Method for discretizing the root and for implementing numerical simulations. **Notes S1** Comparison of modelled auxin concentration trend with experimental DII-VENUS data in the literature. **Notes S2** Evaluation of model sensitivity. Please note: Wiley Blackwell are not responsible for the content or functionality of any supporting information supplied by the authors. Any queries (other than missing material) should be directed to the *New Phytologist* Central Office.
