# Supplementary material for: Spatiotemporal modelling of hormonal crosstalk explains the level and patterning of hormones and gene expression in Arabidopsis thaliana wild-type and mutant roots
Source: New Phytol. 2015 Apr 23;207(4):1110–22. doi: 10.1111/nph.13421 (PMC4539600; doi:10.1111/nph.13421)
Supplement: Supplementary file 1 [file nph0207-1110-sd1.pdf]

New Phytologist **Supporting Information Figs S1–S12; Table S1; Methods S1, S2; Notes S1, S2**

Spatiotemporal modelling of hormonal crosstalk explains the level and patterning of hormones and gene expression in *Arabidopsis thaliana* wild type and mutant roots

Simon Moore, Xiaoxian Zhang, Anna Mudge, James H. Rowe, Jennifer F. Topping, Junli Liu and Keith Lindsey

Article acceptance date: 20 March 2015

The following Supporting Information is available for this article:

Fig. S1 Trend in average root auxin concentration in wild type and mutants.

Fig. S2 Modelling results show that PIN and AUX1 auxin carrier proteins localise predominantly to the plasma membrane in the wild type.

Fig. S3 Cytokinin images and concentration profiles.

Fig. S4 Auxin patterning for different combinations of PIN and AUX1 permeability.

Fig. S5 Modelled auxin concentration profiles for the three different cell types (epidermal, pericycle and vascular cells).

Fig. S6 DII-VENUS response profile measured from the experimental image, compared to the model auxin concentration profile for wild type root.

Fig. S7 Modelling results for PINm transcription rates in wild type.

Fig. S8 Modelling results for patterning of X, downstream of ethylene signalling, and PLSp, POLARIS protein, in wild type.

Fig. S9 Comparison of experimental and modelling PIN2 patterning for wild type and mutants.

Fig. S10 Modelling results for PLSm transcription patterning in wild type.

Fig. S11 Modelling prediction of ethylene patterning is similar to experimental measurements.

Fig. S12 Modelled AUX1 concentration profiles for the three different cell types (epidermal,

pericycle and vascular cells).

Table S1 Model equations and parameter values for the model described in Figs 1 and 2.

Methods S1 Using ImageJ to analyse experimental images.

Methods S2 Method for discretising the root and for implementing numerical simulations.

Notes S1 Comparison of modelled auxin concentration trend with experimental DII-VENUS data in the literature.

Notes S2 Evaluation of model sensitivity.

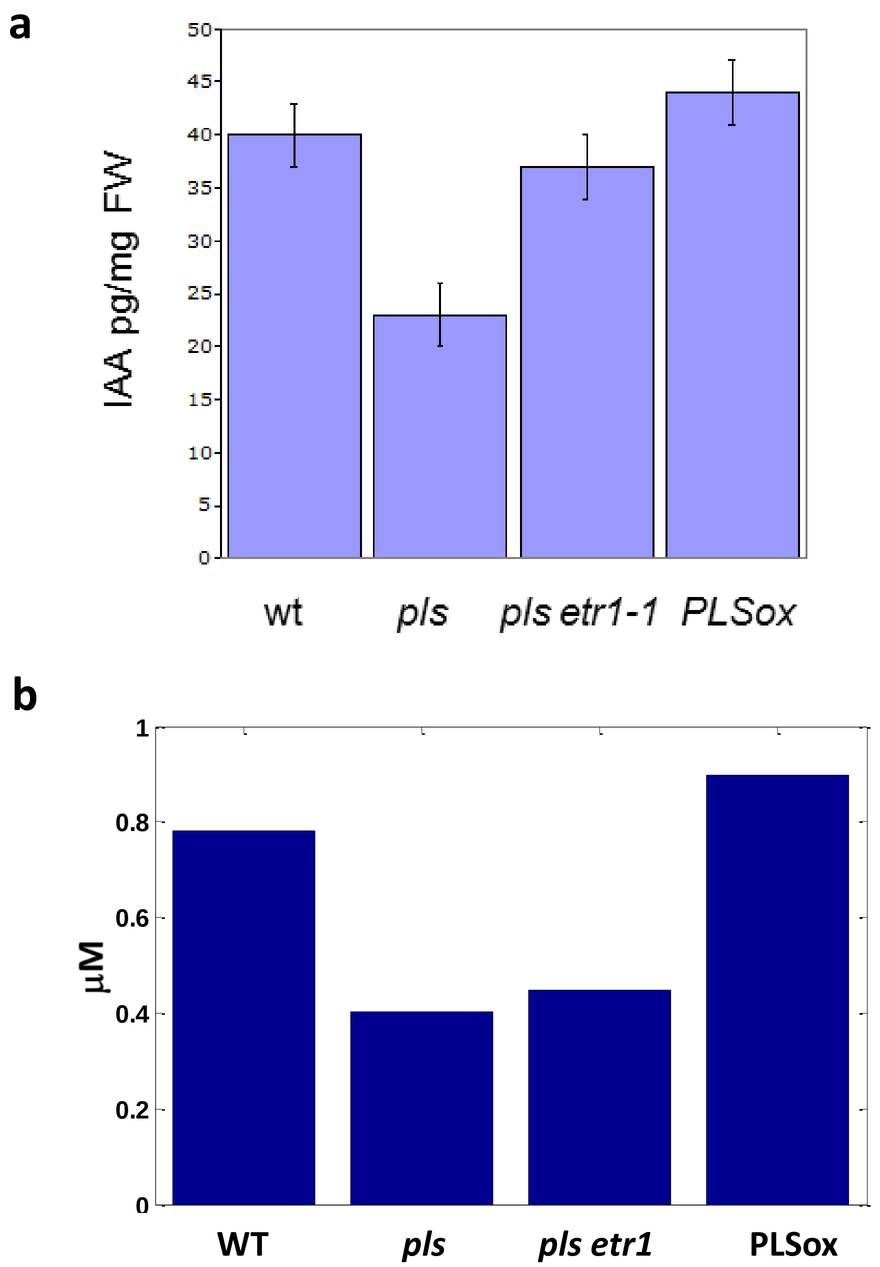

Fig. S1 Trend in average root auxin concentration in wild type and mutants. (a) Experimental results (Fig. 4C, Chilley *et al*., 2006; www.plantcell.org, Copyright American Society of Plant Biologists). (b) Model results. For mutants, the following parameters are used. *pls*: k_6=0.0 s^{-1}; pls etr1: k_6=0.0 s^{-1} , k_{11}=0.025 $\mu M^{-1}s^{-1}$; PLSox: k_6=0.0 45s^{-1}. All other parameters are the same as in Table S1. Error bars represent standard deviations of the mean (n = 3).

Reference

Chilley PM, Casson SA, Tarkowski P, Hawkins N, Wang KL, Hussey PJ, Beale M, Ecker JR, Sandberg GK, Lindsey K. 2006. The POLARIS peptide of Arabidopsis regulates auxin transport and root growth via effects on ethylene signaling. *Plant Cell* **18**: 3058–3072.

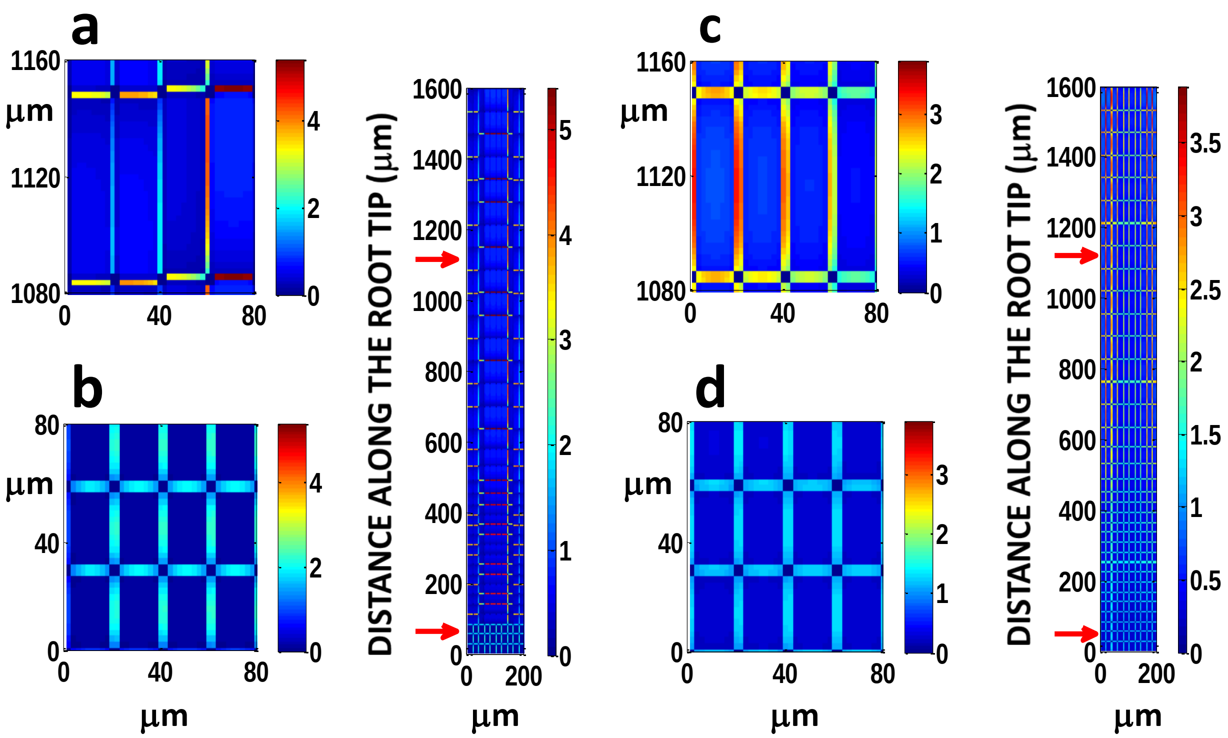

Fig. S2 Modelling results show that PIN and AUX1 auxin carrier proteins localise predominantly to the plasma membrane in the wildtype. All parameters are included in Table S1. (a, b) Localisation of PIN proteins to the plasma membrane at different root locations marked by red arrows. An example of PIN protein polarity is shown in (a). (c, d) Localisation of AUX1 proteins to the plasma membrane at different root locations marked by red arrows.

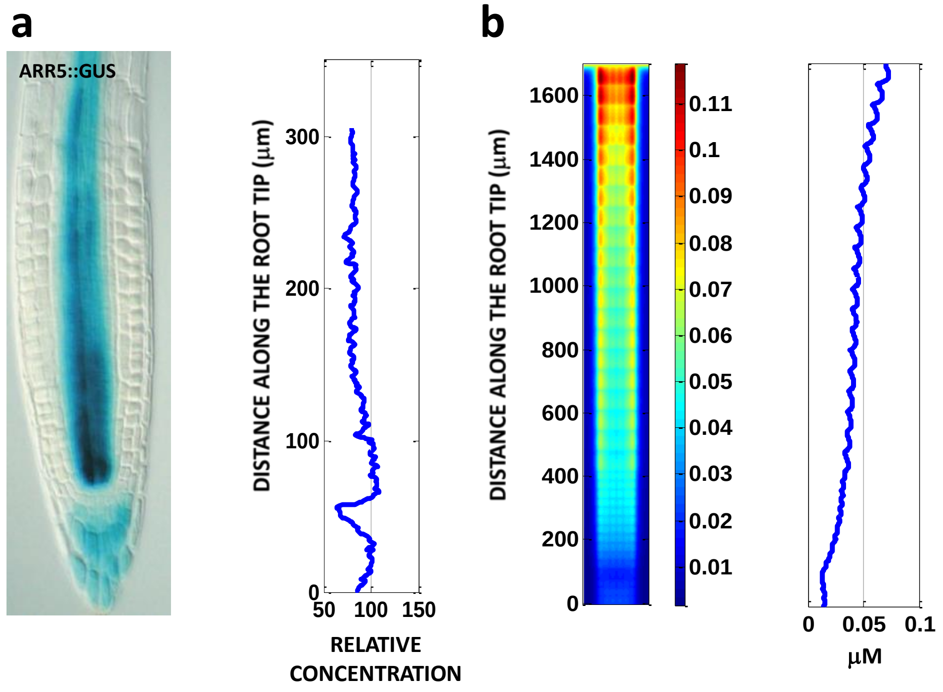

Fig. S3 Cytokinin imaging and concentration profiles. (a) Experimental image (ARR5::GUS reporter) and profile (Werner *et al*., 2003; www.plantcell.org, Copyright American Society of Plant Biologists). (b) Modelling results, image and profile. All parameters are included in Table S1.

Reference

Werner T, Motyka V, Laucou V, Smets R. van Onckelen H, Schmülling T. 2003. Cytokinin-deficient transgenic Arabidopsis plants show multiple developmental alterations indicating opposite functions of cytokinins in the regulation of shoot and root meristem activity. *The Plant Cell* **15**: 2532–2550.

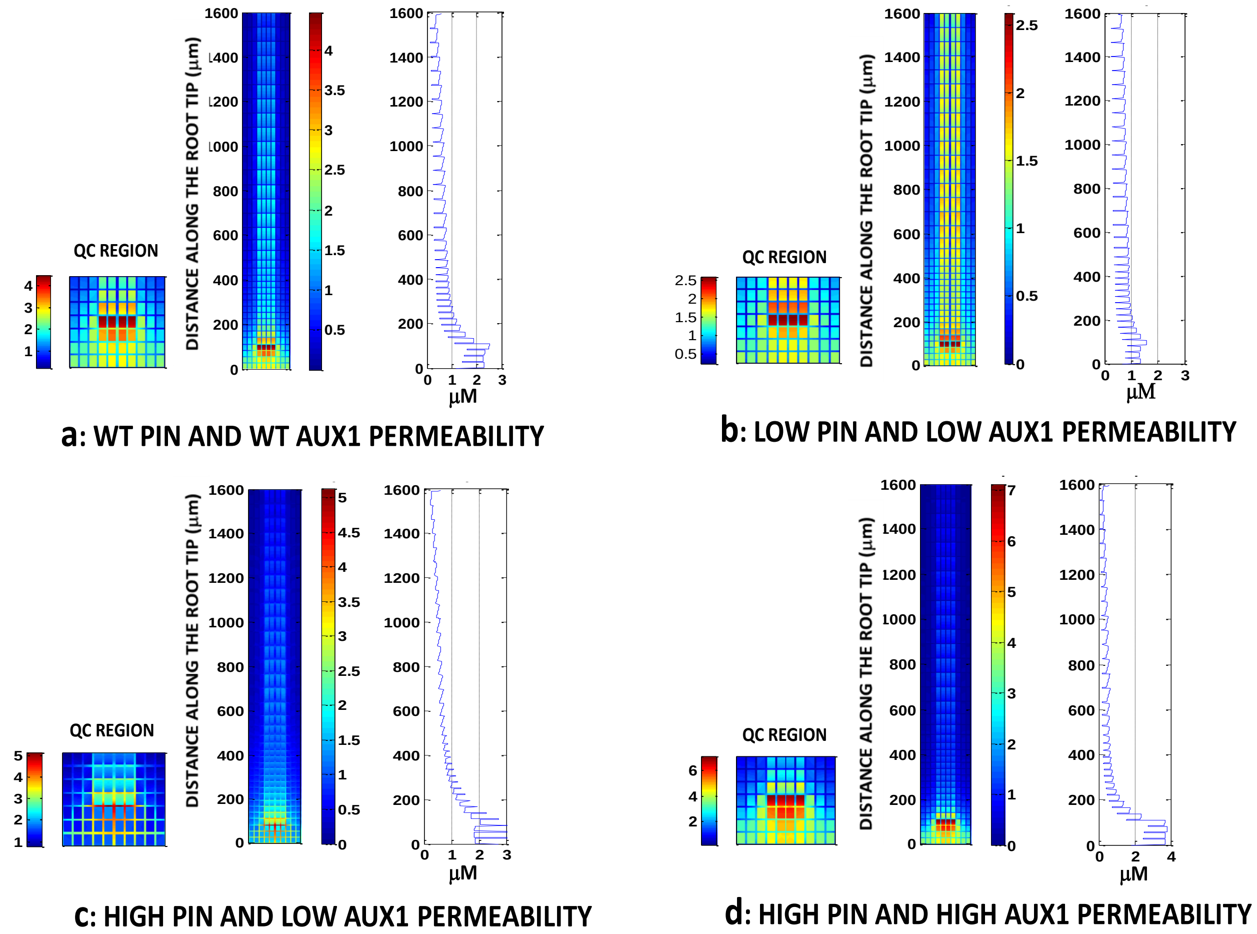

Fig. S4 Auxin patterning for different combinations of PIN and AUX1 permeability.

(a) All the parameters are the same as in Table S1. (b) k_{3b}= 0.2 μm s^{-1} $μM^{-1}$, k_{31}=1.0 μm s^{-1} $μM^{-1}$, all other parameters are the same as in Table S1. (c) k_{3b}= 0.8 μm s^{-1} $μM^{-1}$, k_{31}= 1.0 μm s^{-1} $μM^{-1}$, all other parameters are the same as in Table S1. (d) k_{3b}=0.8 μm s^{-1} $μM^{-1}$, k_{31}=4.0 μm s^{-1} $μM^{-1}$, all other parameters are the same as in Table S1.

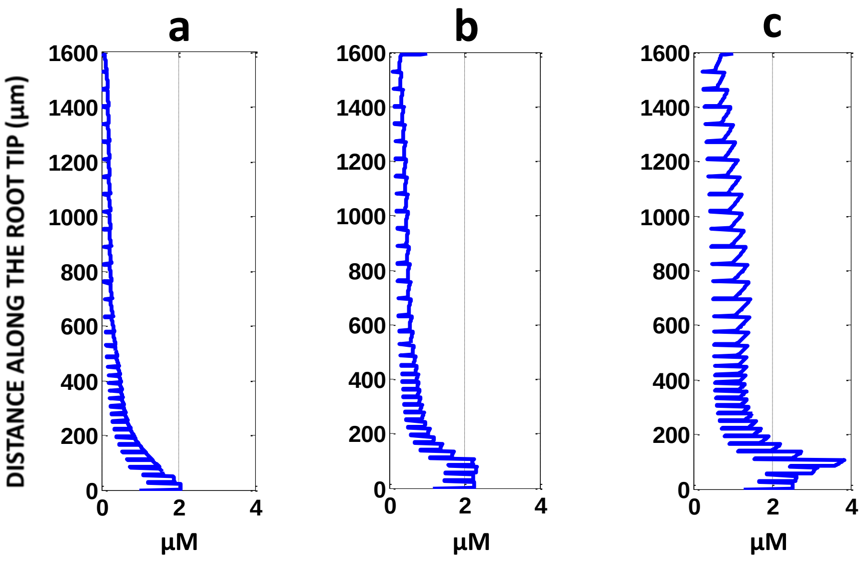

Fig. S5 Modelled auxin concentration profiles for the three different cell types (epidermal, pericycle and vascular cells), showing that the auxin maximum is predominantly established in the central tissues at or close to the QC. (a) epidermal cells; (b) pericycle cells; (c) vascular cells.

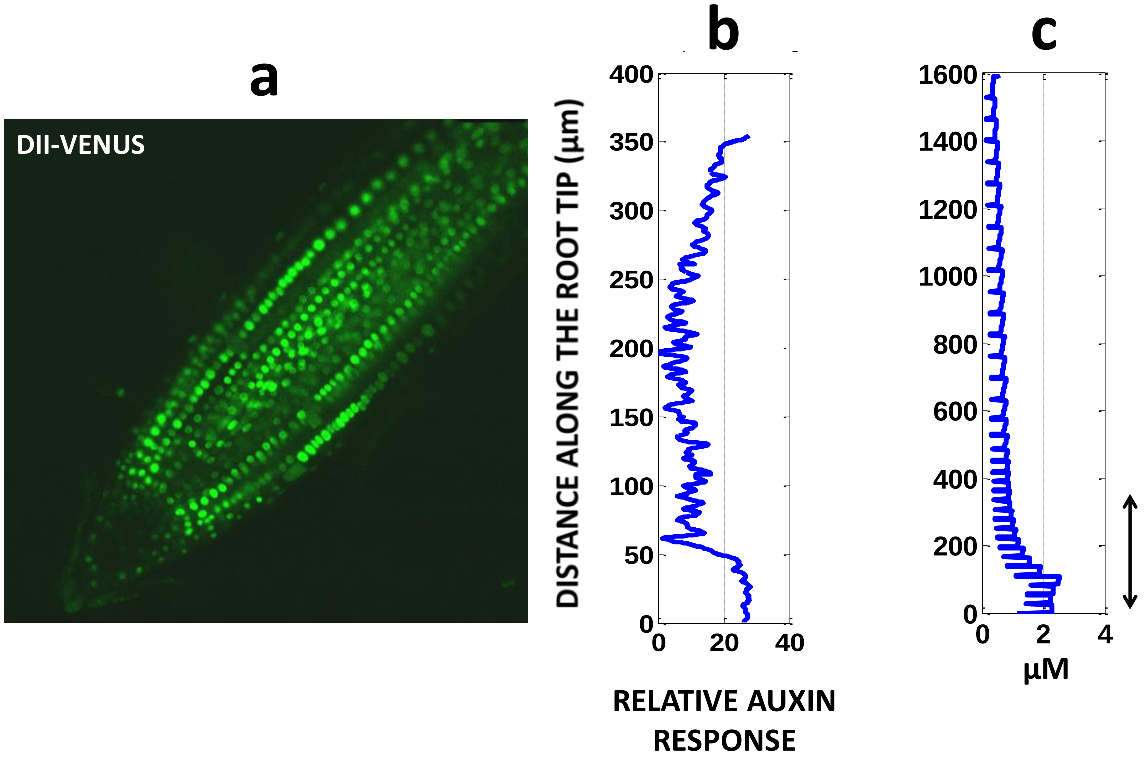

Fig. S6 DII-VENUS response profile measured from the experimental image, compared to the model auxin concentration profile for wildtype root. (a) Experimental image of DII VENUS response. (b) Relative auxin response profile (inverse of DII VENUS signal) derived from the experimental image of DII VENUS response. (c) Modelled auxin concentration profile. The experimental image profile approximately corresponds to the region of the root in the model denoted by the arrow.

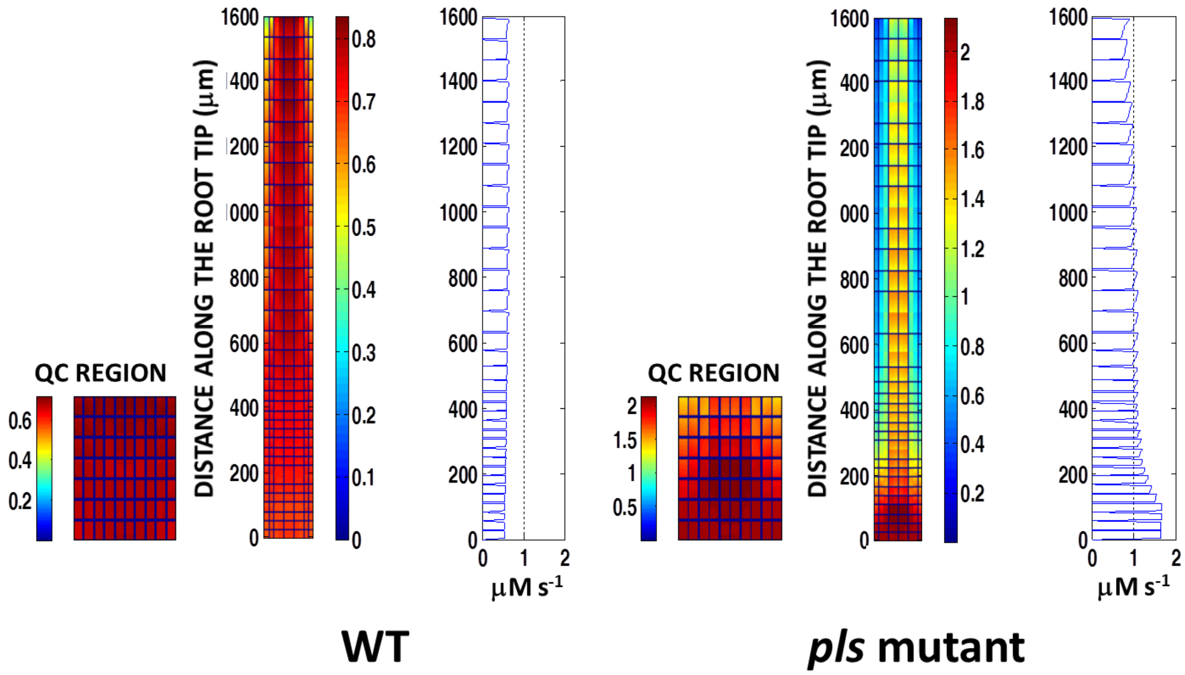

Fig. S7 Modelling results for PINm transcription rates in wild type (all parameters are included in Table S1) and the *pls* mutant (k_6=0.0 s^{-1}, all other parameters are the same as in Table S1).

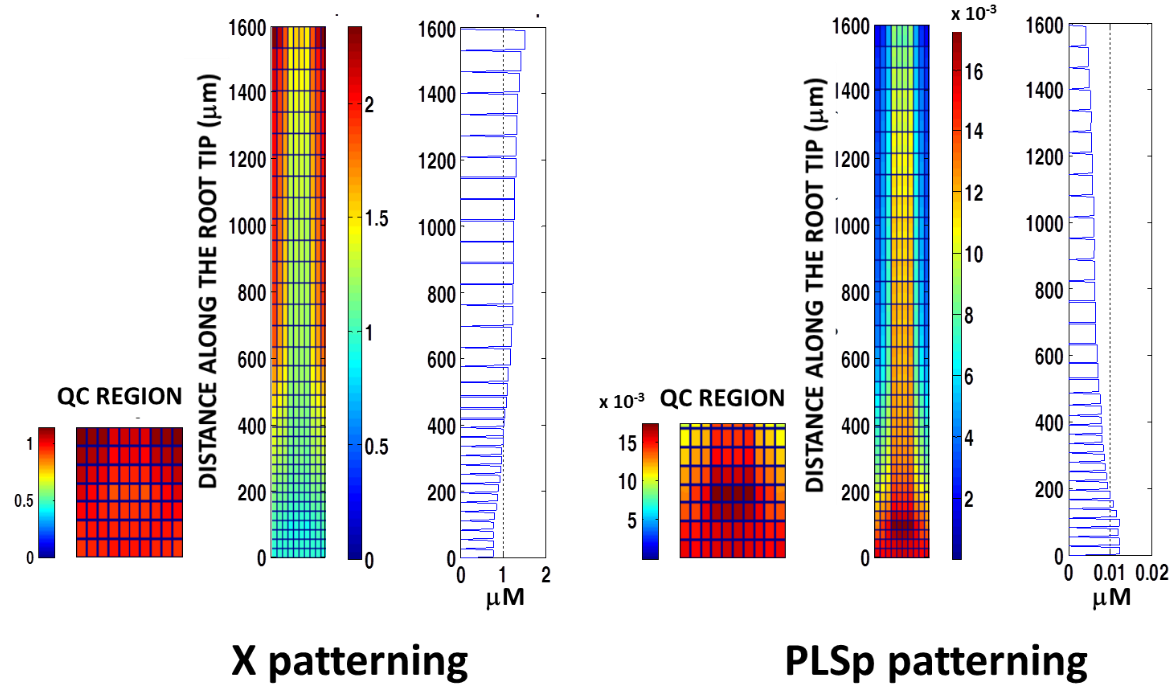

Fig. S8 Modelling results for patterning of X, downstream of ethylene signalling, and PLSp, POLARIS protein, in wildtype. All parameters are included in Table S1.

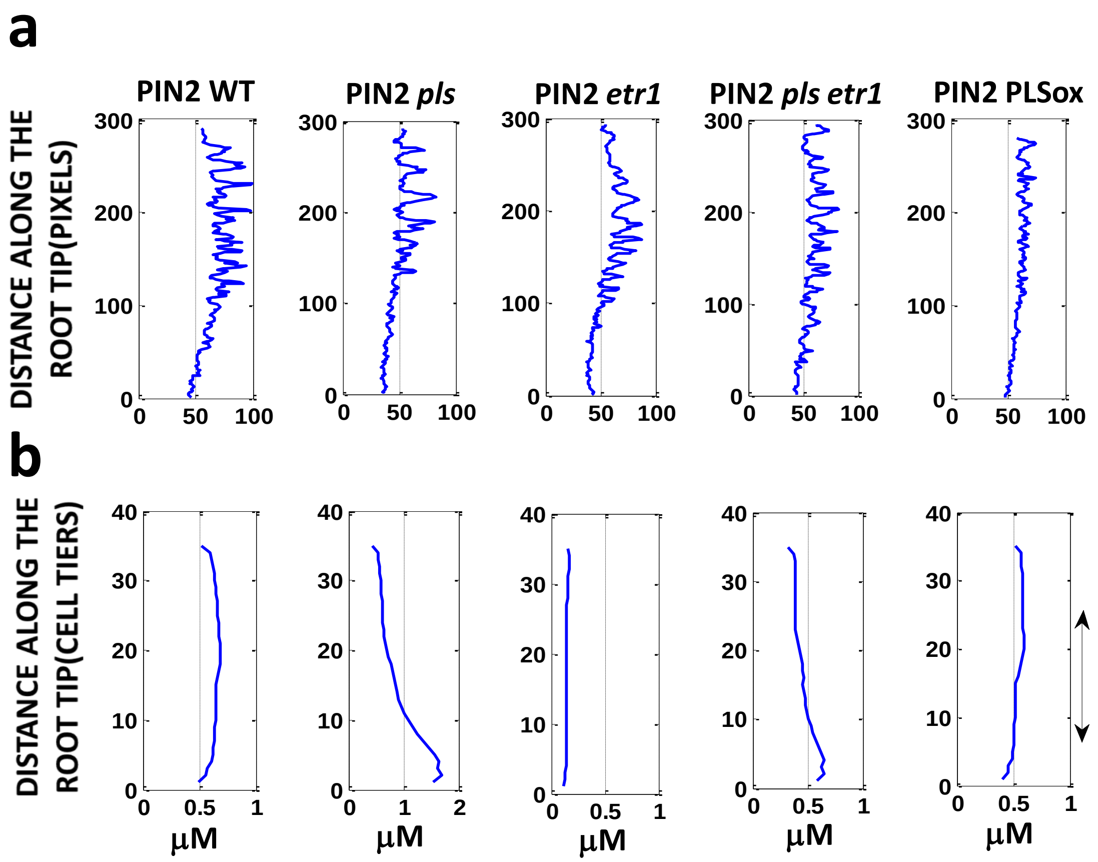

Fig. S9 Comparison of experimental and modelling PIN2 patterning for wildtype and mutant roots. (a) Quantification of PIN2 protein by analysing the experimental images (Fig. 1, Liu *et al*., 2013). (b) Modelling prediction of the patterning of PIN2 protein. Wild type: all parameters are included in Table S1. Mutants: *pls*: k_6=0.0 s^{-1}; *pls etr1*: k_6=0.0 s^{-1}, k_{11}=0.025 $\mu M^{-1}s^{-1}$; PLSox: k_6=0.0 45s^{-1}. All other parameters are the same as in Table S1.

PIN1 proteins localise mainly to the vascular cell files but with a weak signal in the epidermal and cortical tissues, consistent with Blilou *et al*. (2005). PIN1 concentration data were collected for the vascular tissues, plotted and compared to profiles from the vascular and pericycle cell files from the model. The model profile is a plot of the average PIN1 concentration in each cell tier cross-section of the root rather than each grid point since the large difference between PIN concentrations in the plasma membrane and cytosol make grid point plots difficult to read (Fig. 7 in the main text).

The same process was followed for the PIN2 images. The data were captured from the 2 external regions and then combined. However, given the location of the PIN2 proteins in the lateral root cap, epidermis and cortical cells and the tapering root shape it was more difficult to capture data than for the PIN1 proteins in the central vascular region. Fig. S9 shows a) quantification of PIN2 protein by analysing the experimental images (Fig. 1, Liu *et al*. 2013); and b) modelling predictions for the patterning of PIN2 protein. There was a reasonable match in concentration trends in the wild type and PLSox but not for the other mutants. This may be due to the overly simplistic structure of the root model where the region of

PIN2 expression in the model is an even rectangle of cells consisting of the 2 exterior cell files of equal width extending for the full length of the root. In reality, the region of PIN2 expression in the root is not a simple rectangle; it consists of the lateral root cap (which only extends to the EZ zone and is not included in the modelled root structure), and the epidermal and cortical cell files starting about 5 cell tiers above the QC region (Muller *et al*., 1998).

References

Blilou I, Xu J, Wildwater M, Willemsen V, Paponov I, Friml J, Heidstra R, Aida M, Palme K, Scheres B. 2005. The PIN auxin efflux facilitator network controls growth and patterning in Arabidopsis roots. *Nature* **433**: 39-44.

Liu JL, Mehdi S, Topping J, Friml J, Lindsey K. 2013. Interaction of PLS and PIN and hormonal crosstalk in Arabidopsis root development. *Frontiers in Plant Science* **4**: 75.

Muller A, Guan C, Galweiler L, Tanzler P, Huijser P, Marchant A, Parry G, Bennett M, Wisman E, Palme K. 1998. AtPIN2 defines a locus of Arabidopsis for root gravitropism control. *EMBO Journal* **17**: 6903–6911.

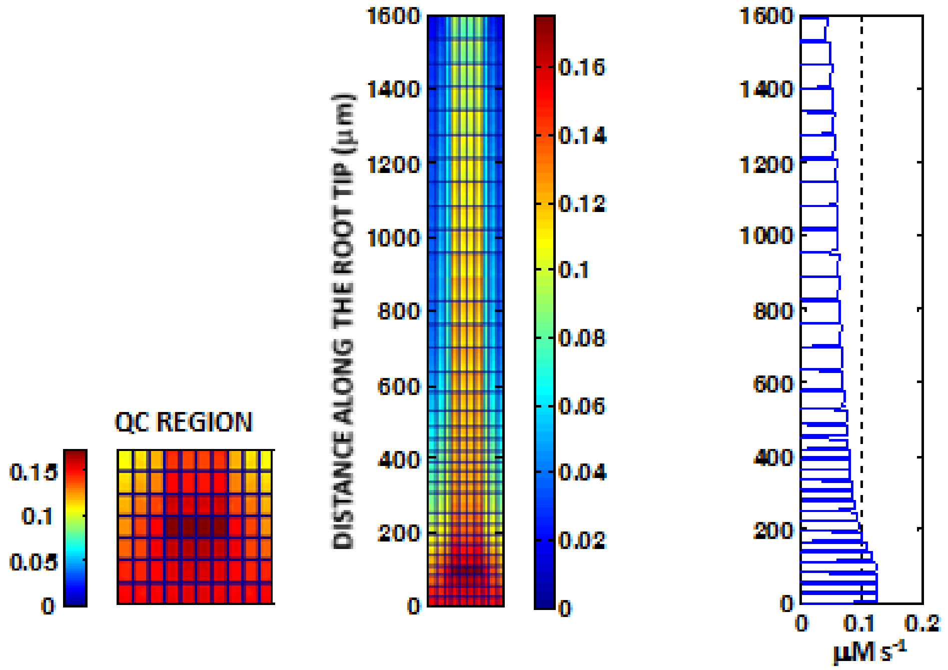

Fig. S10 Modelling results for PLSm transcription patterning in wild type. All parameters are the same as in Table S1.

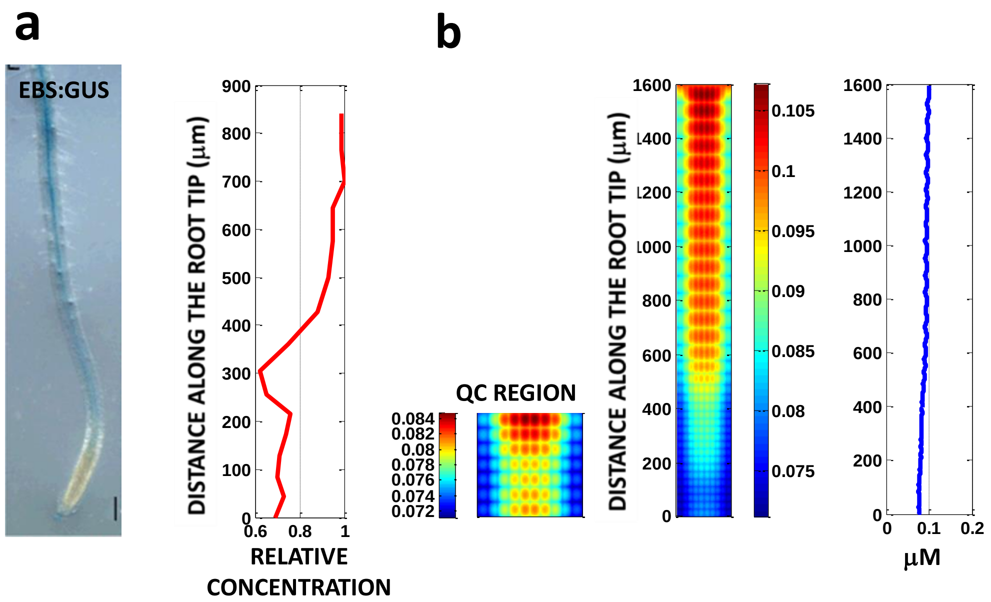

Fig. S11 Modelling prediction of ethylene patterning is similar to experimental measurements. (a) Ethylene profiles calculated from an experimental image (Fig. 5E, Martin-Rejano *et al*., 2011, with permission). (b) Modelling results.

References

Martin-Rejano EM, Camacho-Cristoval JJ, Herrera-Rodriguez MB, Rexach J, Navarro-Gochicoa MT and Gonzales-Fontes A. 2011. Auxin and ethylene are involved in the responses of root system architecture to low boron supply in Arabidopsis. *Physiologia Plantarum* **142**: 170–178.

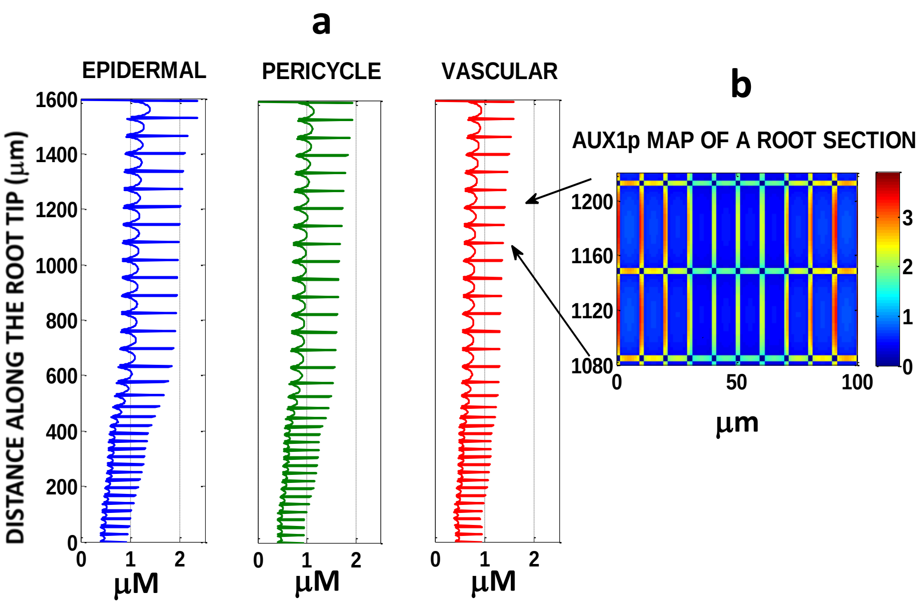

Fig. S12 Modelled AUX1 concentration profiles for the three different cell types (epidermal, pericycle and vascular cells). (a) Modelled AUX1 concentration profiles for the three different types (epidermal, pericycle and vascular cells). (b) Blow-up of the area marked by arrows, showing details of the lateral distribution of AUX1 protein. This figure shows that AUX1 level increases proximally in the epidermal cells and also that epidermal, pericycle and vascular cells have high, medium and low AUX1 protein levels, respectively.

Table S1 Model equations and parameter values for the model described in Figs 1 and 2 and references used for deriving the equations and parameters.

A: Rate equations for biosynthesis, decay, activation and inactivation (Those reaction rates in Fig. 1d are summarised in this Table)		
**Species**	**Rate equations and parameter values**	**Notes**
**AUXIN**		
V1 Background biosynthesis	k_2	Only in the cytosol
V2 Variable biosynthesis	$\frac{k_{2a}[ET]}{(k_{2d}+k_{2e}[ET])(1+[CK]/k_{2b})} \frac{[PLSp]}{(k_{2c}+[PLSp])}$	Only in the cytosol
V3 Decay	$k_3[Auxin]$	In the cytosol and cell walls
Parameters	k2 = 0.001 μM s^{-1}; k2a = 0.025 s^{-1}; k2b=1.0 μM; k2c = 0.01 μM; k2d = 1.0; k2e = 0.0 μM^{-1}; k3 = 0.002 s^{-1}	
References	Eklof *et al*., 1997; Liu *et al*., 2010; Ljung, 2013; Stepanova *et al*., 2007; Swarup *et al*., 2007; Tivendale *et al*., 2014; Zhao, 2010.	
**Ra***		
V4 Rate of activation	$k_4[Aux1m](RaT-Ra^*)$	Only in the cytosol. The receptor switches between active and inactive with the total RaT remaining constant.
V5 Rate of inactivation	$k_5 Ra^*$	Only in the cytosol
Parameters	k4 = 1.0 μM^{-1} s^{-1}; k5 = 1.0 s^{-1}	
References	Liu *et al*., 2010; Ljung, 2013; Mockaitis & Estelle, 2008; Vanneste & Friml, 2009.	
**PLSm**		
V6 Rate of transcription	$\frac{k_6[Ra^*]}{1+\frac{[ET]}{k_{6a}}}$	Only in the cytosol
V7 Rate of decay	$k_7[PLSm]$	Only in the cytosol

Parameters	k6 = 0.03 s^{-1}; k6a = 0.2 μM; k7 = 1.0 s^{-1}; for *pls* null mutant k6 = 0.0 s^{-1}; for PLSox k6 = 0.045 s^{-1}	
References	Casson *et al*., 2002; Chilley *et al*., 2006; Liu *et al*., 2010; Ljung, 2013; Mockaitis & Estelle, 2008; Vanneste & Friml, 2009.	
**PLSp**		
V8<br>Rate of translation	$k_8[PLSm]$	Only in the cytosol
V9<br>Rate of decay	$k_9[PLSp]$	Only in the cytosol
Parameters	k8 = 1.0 s^{-1}; k9 = 1.0 s^{-1}	
References	Casson *et al*., 2002; Chilley *et al*., 2006; Liu *et al*., 2010; Ljung, 2013; Mockaitis & Estelle, 2008; Vanneste & Friml, 2009.	
**Re^***		
V10<br>Rate of activation	$(k_{10}+k_{10a}[PLSp])([\mathrm{Re}T]-[\mathrm{Re}^*])$	Only in the cytosol. The receptor switches between active and inactive with the total ReT remaining constant.
V11<br>Rate of inactivation	$k_{11}[\mathrm{Re}^*][ET]$	Only in the cytosol
Parameters	k10=0.0003 s^{-1}; k10a=5.0 μM^{-1} s^{-1}; k11=4.0 μM^{-1} s^{-1}; for the *etr1* mutant k11 = 0.025 μM^{-1} s^{-1}	
References	Diaz & Alvarez-Buylla, 2006; Liu *et al*., 2010; Wang *et al*., 2002.	
**ET**		
V12<br>Rate of biosynthesis	$k_{12}+k_{12a}(\frac{[Auxin]}{(k_{12b}+k_{12d1}[Auxin])}\ \frac{[CK]}{(k_{12c}+k_{12d2}[CK])})$	Only in the cytosol. Michaelis Menten kinetics for the rate of biosynthesis regulated by Auxin and CK.
V13<br>Rate of decay	$k_{13}[ET]$	In the cytosol and cell walls
Parameters	k12 = 0.1 μM s^{-1}; k12a = 0.1 μM^{-1} s^{-1}; k12b = 0.1;<br>k12c = 0.1; k12d1 = 1.0 μM^{-1}; k12d2 = 1.0;<br>k13 = 1.0 s^{-1}	
References	Liu *et al*., 2010; Vogel *et al*., 1998;<br>Stepanova *et al*., 2007; Tanimoto *et al*., 1995.	

**CTR1***		
V14<br>Rate of activation	$k_{14}[\mathrm{Re}^*]([CTR1T]-[CTR1^*])$	Only in the cytosol. The receptor switches between active and inactive with the total CTR1T remaining constant.
V15<br>Rate of inactivation	$k_{15}[CTR1^*]$	Only in the cytosol
Parameters	k14 = 3.0 μM^{-1} s^{-1}; k15 = 0.085 s^{-1}	
References	Diaz & Alvarez-Buylla, 2006; Liu *et al*., 2010; Wang *et al*., 2002.	
**X**		
V16<br>Rate of pathway activation	$k_{16}-k_{16a}[CTR1^*]$	Only in the cytosol. Pathway inhibition is regulated by active CTR1.
V17<br>Rate of pathway inactivation	$k_{17}[X]$	Only in the cytosol
Parameters	k16 = 0.3 μM s^{-1}; k16a = 1.0 s^{-1}; k17 = 0.1 s^{-1}	
References	Diaz & Alvarez-Buylla, 2006; Liu *et al*., 2010.	
**CK**		
V18<br>Rate of biosynthesis	$\dfrac{k_{18a}}{1+\dfrac{[Auxin]}{k_{18}}}$	Only in the cytosol in the pericylce and vascular cell files in the MZ and EZ regions
V19<br>Rate of decay	$k_{19}[CK]$	In the cytosol and cell walls
Parameters	k18 = 0.1 μM; k18a = 1.0 μM s^{-1}; k19 = 1.0 s^{-1}	
References	Liu *et al*., 2010; Nordstrom *et al*., 2004.	
**PINm**		
V20<br>Rate of transcription	$\dfrac{k_{20a}[X][Auxin]}{(k_{20b}+[CK])(k_{20c}+[Auxin])}$	Only in the cytosol
V21<br>Rate of decay	$k_{21a}[PINm]$	Only in the cytosol
Parameters	k20a = 0.8 μM s^{-1}; k20b = 1.0 μM; k20c = 0.3 μM;<br>k21a = 1.0 s^{-1}	
References	Chandler, 2009; Liu *et al*., 2010; Liu *et al*.,	

	2013; Nordstrom *et al*., 2004; Paciorek *et al*., 2005; Ruzicka *et al*., 2007, 2009; Swarup *et al*., 2007. Vanneste & Friml, 2009.	
**PINp**		
V22 Rate of translation	$k_{22a}[PINm]$	Only in the cytosol
V23 Rate of decay	$k_{23a}[PINp]$	In the cytosol and cell walls
Parameters	k22a = 1.0 s^{-1}; k23a = 0.75 s^{-1}	
References	Chandler, 2009; Liu *et al*., 2010; Liu *et al*., 2013; Nordstrom *et al*., 2004; Paciorek *et al*., 2005; Ruzicka *et al*., 2007, 2009; Swarup *et al*., 2007. Vanneste & Friml, 2009.	
**AUX1m**		
V26 Rate of transcription	$k_{1a}[X]$	Only in the cytosol.
V27 Rate of decay	$k_{26}[Aux1m]$	Only in the cytosol
Parameters	k1a = 0.8 μM s^{-1}; k26=1.0s^{-1}	
References	Ruzicka *et al*., 2007; This work.	
**AUX1p**		
V28 Rate of translation	$k_{27}[Aux1m]$	Only in the cytosol
V29 Rate of decay	$k_{28}[Aux1p]$	In the cytosol and cell walls
Parameters	k27 = 1.0 s^{-1}; k28 = 1.0 s^{-1}	
References	This work	
**B: Equations for recycling of PIN and AUX1 to and from the plasma membrane**		
**Species**	**Rate equations and parameter values**	**Notes**
**PINp**		
V24 Rate of localisation of PINp to the plasma membrane	$k_{24a}(i)[PINpi]$	[PINpi] is the PIN concentration at the cytosolic GP. k24a(i) depends on the property of the NN plasma membrane GP (i) as shown in the parameter values.

V25<br>Rate of removal of PINp from the plasma membrane	$$\frac{k_{25a}\,[PINpm]}{(1+[Auxin]_0\,/\,k_{25b})}$$	[PINpm] is the PIN concentration at the plasma membrane GP. $[Auxin]_0$ is the auxin concentration at the NN cytosolic GP
Parameters	Localising PIN proteins to plasma membrane:<br>Low: k24a(1)=1.0 s^{-1}; medium: k24a(2) = 5.0 s^{-1}; high: k24a(3)=20.0 s^{-1};<br>K25a = 1.0 s^{-1}; k25b = 1.0 μM	
References	Grieneisen *et al*., 2007; Liu *et al*., 2013; Paciorek *et al*., 2005; This work.	
**AUX1p**		
V30<br>Rate of localisation of AUX1p to the plasma membrane	$k_{29}[Aux1pi]$	[AUX1pi] is the AUX1 concentration at the cytosolic GP.
V31<br>Rate of removal of AUX1p from the plasma membrane	$k_{30}[Aux1pm]$	[AUX1pm] is the AUX1 concentration at the plasma membrane GP.
Parameters	k29 = 10.0 s^{-1}; k30 = 1.0 s^{-1}	
References	This work	

C: Species flux between nearest neighbour (NN) grid points A to B

Species	A →	B	Flux equation	Notes
**Auxin (diffusion)**	0	0	$Auxindiff\ (cell)\ ([Auxin]_A - [Auxin]_B)/\Delta x$	Diffusion in the cytosol
	1or 2 or 3	1or 2 or 3	$Auxindiff\ (wall)\ ([Auxin]_A - [Auxin]_B)/\Delta x$	Diffusion in the cell wall
	4 or 5	any	No diffusion from GP 4 and 5	Border to cytosol
	any	4 or 5	No diffusion to GP 4 and 5	Cytosol to border
Parameters			Auxindiff(cell) = 220 $\mu m^2\ s^{-1}$<br>Auxindiff(wall) = 220 $\mu m^2\ s^{-1}$; Δx = 2.0μm	
References			Kramer *et al*., 2011; Rutschow *et al*., 2011; This work.	
**Auxin efflux (permeability)**	0	1,2 or 3	$p(A,B)k_{3b}[PINp]_B[Auxin]_A$	Efflux from the cell<br>p(A,B) = 1

	0	4	Above equation but zero flux	p(A,B) = 0
	0	5	Above equation and flux occurs	Efflux from the root to the shoot. p(A,B) = 1
Parameters			p(A,B) is a switch determining if permeability can occur from A to B and is = 0 or 1. Δx = 2.0μm; k3b = 0.4μm s^{-1} μM^{-1}	
References			Kramer *et al*., 2011; This work	
**Auxin influx (permeability)**	1,2 or 3	0	$p(A,B)k_{31}[AUX1]_A[Auxin]_A$	Influx into the cell p(A,B) = 1
	4	0	$p(A,B)\dfrac{k_{32a}[Auxin]_A}{1+[X]_B/k_{32b}}$	Shoot to root influx p(A,B) = 1
	5	0	Same equation as 4 to 0 but zero flux since p(A,B) = 0	Shoot to root influx p(A,B) = 0
Parameters			p(A,B) is a switch determining if permeability can occur from A to B and is = 0 or 1 dx = 2.0μm (scaling constant); k31 = 2.0μm s^{-1} μM^{-1}; k32a = 10μm s^{-1}; k32b = 0.1 μM	
References			Chilley *et al*., 2006; Kramer, 2004; Rutschow *et al*., 2014; Suttle, 1988; This work.	
**ET (diffusion)**	0, 1, 2, 3, 4 or 5	0, 1, 2, 3, 4 or 5	$ETdiff\ ([ET]_A-[ET]_B)/\Delta x$	ET diffuses between all GP of the same or different types whether in the cytosol or cell wall with the same diffusion coefficient
Parameters			ETdiff = 600 μm^2 s^{-1}; Δx = 2.0μm	
References			This work	
**CK (diffusion)**	0, 1, 2, 3, 4 or 5	0, 1, 2, 3, 4 or 5	$CKdiff\ ([CK]_A-[CK]_B)/\Delta x$	CK diffuses between all GP of the same or different types whether in the cytosol or cell wall with the same diffusion coefficient
Parameters			CKdiff = 220 μm^2 s^{-1}; Δx = 2.0μm	
References			Mellor & Bishopp, 2014; This work	
**All other species (diffusion)**	0	0	$Otherdiff\ ([Other]_A-[Other]_B)/\Delta x$	Diffuse within the cytosol only and do not cross the PM

				and enter the cell wall (for PINp and AUX1p recycling see Table 4)
Parameters			Otherdiff = 220 μm^2 s^{-1}; Δx = 2.0μm	
References			This work	

References

Casson SA, Chilley PM, Topping JF, Evans IM, Souter MA, Lindsey K. 2002. The *POLARIS* gene of Arabidopsis encodes a predicted peptide required for correct root growth and leaf vascular patterning. *The Plant Cell* **14**: 1705–1721.

Chandler JW. 2009. Auxin as compère in plant hormone crosstalk. *Planta* **231**: 1–12.

Chilley PM, Casson SA, Tarkowski P, Hawkins N, Wang KL, Hussey PJ, Beale M, Ecker JR, Sandberg GK, Lindsey K. 2006. The POLARIS peptide of Arabidopsis regulates auxin transport and root growth via effects on ethylene signaling. *The Plant Cell* **18**: 3058–3072.

Diaz J, Alvarez-Buylla E. 2006. A model of the ethylene signalling pathway and its gene response in *Arabidopsis thaliana*: pathway cross-talk and noise-filtering properties. *Chaos* **16**: 02311201–02311216.

Eklof S, Astot C, Blackwell J, Moritz T, Olsson O, Sandberg G. 1997. Auxin–cytokinin interactions in transgenic tobacco. *Plant Cell Physiology* **38**: 225–235.

Grieneisen VA, Xu J, Marée AFM, Hogeweg P, Scheres B. 2007. Auxin transport is sufficient to generate a maximum and gradient guiding root growth. *Nature* **449**: 1008–1013.

Kramer EM. 2004. PIN and AUX/LAX proteins: their role in auxin accumulation. *Trends in Plant Science* **9**: 578–582.

Kramer EM, Rutschow HL, Mabie SS. 2011. AuxV: A databaseof auxin transport velocities. *Trends in Plant Science* **16**: 461–463.

Liu JL, Mehdi S, Topping J, Tarkowski P, Lindsey K. 2010. Modelling and experimental analysis of hormonal crosstalk in Arabidopsis. *Molecular Systems Biology* **6**: 373.

Liu JL, Mehdi S, Topping J, Friml J,Lindsey K. 2013. Interaction of PLS and PIN and hormonal crosstalk in Arabidopsis root development. *Frontiers in Plant Science* **4**: 75.

Ljung K. 2013. Auxin metabolism and homeostasis during plant development. *Development* **140**: 943–950.

Mellor N, Bishopp A. 2014. The innermost secrets of root development. *Science* **345**: 622–623.

Mockaitis K, Estelle M. 2008. Auxin receptors and plant development: a new signaling paradigm. *Annual Review of Cell and Developmental Biology* **24**: 55–80.

Nordstrom A, Tarkowski P, Tarkowska D, Norbaek R, Astot C, Dolezal K, Sandberg G. 2004. Auxin regulation of cytokinin biosynthesis in *Arabidopsis thaliana*: a factor of potential importance for auxin–cytokinin-regulated development. *Proceedings of the National Academy of Sciences, USA* **101**: 8039–8044.

Paciorek T, Zazimalova E, Ruthardt N, Petrasek J, Stierhof YD, Kleine-Vehn J, Morris DA, Emans N, Jürgens G, Geldner N *et al.* 2005. Auxin inhibits endocytosis and promotes its own efflux from cells. *Nature* **435**: 1251–1256.

Rutschow HL, Baskin TI, Kramer EM. 2011. Regulation of solute flux through plasmodesmata in the root meristem. *Plant Physiology* **155**: 1817–1826.

Rutschow HL, Baskin TI, Kramer EM. 2014. The carrier AUXIN RESISTANT (AUX1) dominates auxin flux into Arabidopsis protoplasts. *New Phytologist* **204**: 536–544.

Ruzicka K, Ljung K, Vanneste S, Podhorska R, Beeckman T, Friml J, Benkova E. 2007. Ethylene regulates root growth through effects on auxin biosynthesis and transport-dependent auxin distribution. *The Plant Cell* **19**: 2197–2212.

Ruzicka K, Simásková M, Duclercq J, Petrásek J, Zazímalová E, Simon S, Friml J, Van Montagu MC, Benková E. 2009. Cytokinin regulates root meristem activity via modulation of the polar auxin transport. *Proceedings of the National Academy of Sciences, USA* **106**: 4284–4289.

Stepanova AN, Jun J, Likhacheva AV, Alonso JM. 2007. Multilevel interactions between ethylene and auxin in Arabidopsis roots. *Plant Cell* **19**: 2169–2185.

Suttle JC. 1988. Effect of ethylene treatment on polar IAA transport, net IAA uptake and specific binding of N-1-naphthylphthalamicacid in tissues and microsomes isolated from etiolated pea epicotyls. *Plant Physiology* **88**: 795–799.

Swarup R, Perry P, Hagenbeek D, Van Der Straeten D, Beemster GTS, Sandberg G, Bhalerao R, Ljung K, Bennett MJ. 2007. Ethylene upregulates auxin biosynthesis in Arabidopsis seedlings to enhance inhibition of root cell elongation. *The Plant Cell* **19**: 2186–2196.

Tanimoto M, Roberts K, Dolan L. 1995. Ethylene is a positive regulator of root-hair development in *Arabidopsis thaliana*. *The Plant Journal* **8**: 943–948.

Tivendale ND, Ross JJ, Cohen JD. 2014. The shifting paradigms ofauxin biosynthesis. *Trends in Plant Science* **19**: 44–51.

Vanneste S, Friml J. 2009. Auxin: a trigger for change in plant development. *Cell* **136**: 1005–1016.

Vogel JP, Woeste KE, Theologis A, Kieber JJ. 1998. Recessive and dominant mutations in the ethylene biosynthetic gene ACS5 of Arabidopsis confer cytokinin insensitivity and ethylene overproduction, respectively. *Proceedings of the National Academy of Sciences, USA* **95**: 4766–4771.

Wang KL, Li H, Ecker JR. 2002. Ethylene biosynthesis and signaling networks. *The Plant Cell* **14** (suppl.): S131–S151.

Zhao Y. 2010. Auxin biosynthesis and its role in plant development. *Annual Review of Plant Biology* **61**: 49–64.

Methods S1 Using ImageJ to analyse experimental images

In this work, experimental images were analysed using ImageJ (http://imagej.nih.gov/ij). With ImageJ, it is possible to define regions on an image (such as the vascular cell cylinder in Fig. A below) and determine an estimate for the relative hormone response or protein concentration profiles by measuring the relative signal intensity along the selected region. Regions were defined by a series of consecutive rectangles which progressively diminished in size towards the distal end of the root tip. Relative signal intensity data were collected from each rectangle and concatenated to give a relative hormone response or protein concentration profile for the selected cell files derived from an original experimental image. For example, Fig. A below shows a PIN2 fluorescent image (Liu *et al*., 2013). Using ImageJ, the epidermal and vascular cell files were defined by rectangles, and the relative wt PIN2 concentration profiles for each cell file were extracted. The data acquired were plotted using MATLAB (Fig. A below).

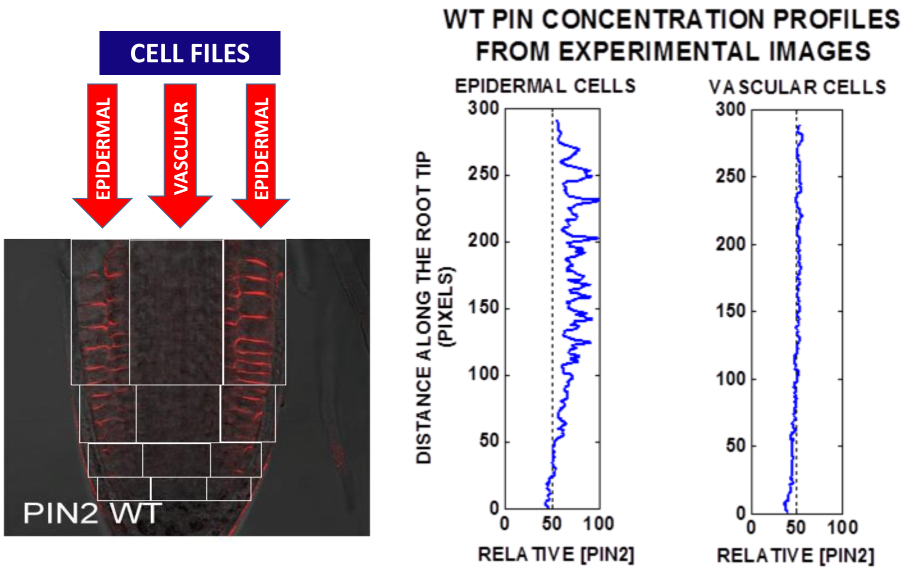

Fig. A Relative PIN2 concentration profiles for epidermal and vascular cell files derived from an experimental image (Liu *et al*., 2013) using ImageJ

Reference

Liu JL, Mehdi S, Topping J, Friml J, Lindsey K. 2013. Interaction of PLS and PIN and hormonal crosstalk in Arabidopsis root development. *Frontiers in Plant Science* **4**: 75.

Methods S2 Method for discretising the root and for implementing numerical simulations

Root structure

We defined a root structure following the Grieneisen *et al*. (2007) model (Fig. A below). The root is 10 cells wide with 4 epidermal cell files, 2 border/pericycle cell files and 4 vascular cell files, with 3 distal tiers of columella cells (Fig. A below). The root is 35 cells in length, including 3 tiers of columella cells, 12 tiers in the meristematic zone (MZ) and 20 tiers in the elongation zone (EZ). Cell wall thickness is 2 µm, and the width of all cells is 20 µm.

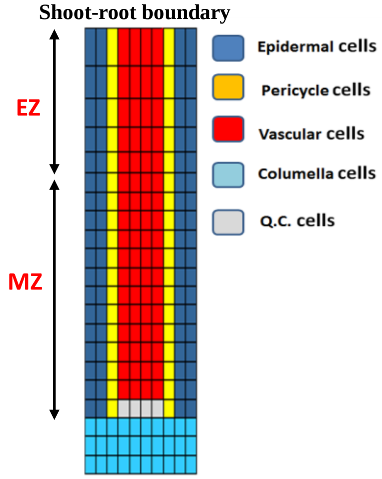

Fig. A Root structure. MZ, meristematic zone; EZ, elongation zone; QC, quiescent centre.

Cell length varies along the longitudinal root axis. Cells in the columella and MZ regions are 28 µm long, including the cell walls. Since cortical cell lengths in the transition zone were shown to increase at a relatively constant rate from 28 µm to 64 µm over a root distance of approximately 280 µm in 10 d-old plants (Beemster & Baskin, 1998), the Grieneisen *et al*. (2007) root structure was modified by increasing cell lengths at the same rate from 28 µm at the proximal end of the MZ, by 15% per cell tier, to a length of 64 µm over 7 cell tiers into the EZ. The length of these 7 cell tiers was 28 µm, 32 µm, 36 µm, 42 µm, 48 µm, 56 µm and 64 µm, to give an overall root tip length of 1594 µm.

To facilitate the calculation of average concentrations in different parts of the root, 3 cell types were established. Cell type 1 was defined as the 4 epidermal cell files along the entire root length (i.e. including some columella cells); type 2 cells are the 2 pericycle files (including some columella cells); and type 3 cells are the 4 vascular cell files (including the quiescent centre and some columella cells).

Grid point representation of the root

In the model the root is divided up into 2 µm by 2 µm areas, each of which is represented by a grid point (GP, Fig. B below). Each GP can have different properties, depending on its location in the root, and is assigned a numerical property code. The root is therefore represented by a root map which is a 100 by 797 matrix of GP values. For modelling purposes, the plasma membrane

is included in cell wall GPs and the code for each cell wall GP can vary depending on the property of its associated plasma membrane. The properties assigned to each GP allow rules to be established governing such processes as species biosynthesis, decay, activation or inactivation, or species flux between adjacent GPs. Equations are set up to govern the flux between each GP and its 4 nearest neighbour (NN) GPs located to the N, S, E or W (Fig. B below). For example all species can diffuse within the cytosol but only the hormones can cross the plasma membrane (PM) into the cell wall. Cytokinin (CK) and ethylene (ET) can diffuse across the PM while auxin crosses via PIN and AUX1 carrier proteins. Once within the cell walls, the hormones can diffuse between NN GPs. PIN and AUX1 proteins can cycle between the cytosol and the NN cell wall GP but cannot diffuse between adjacent cell wall GPs. Individual kinetic equations and parameter values for these processes are described in Table S1.

a **b**

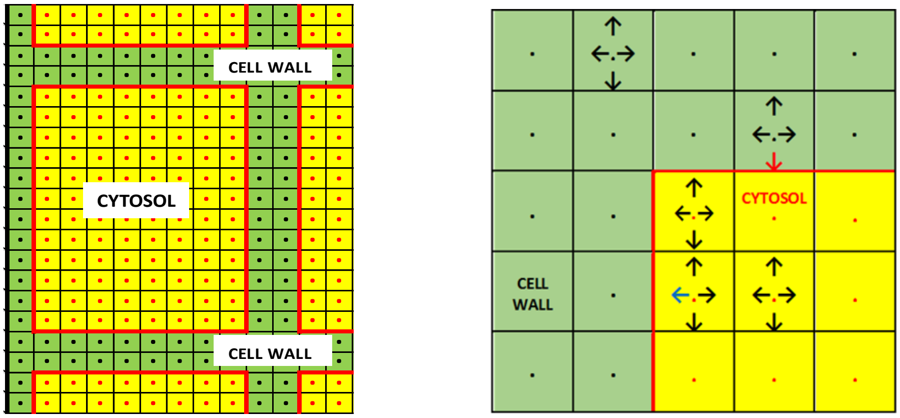

Fig. B Grid point representation of a cell in the MZand example of flux between nearest neighbour (NN) grid points. Green, cell walls; yellow, cytosol; red line, plasma membrane; red dots, grid points in the cytosol; black dots, grid points in the cell wall; black arrows, diffusion between NN; red arrow, auxin cell influx mediated by AUX1 carrier protein; blue arrow, auxin cell efflux mediated by PIN carrier protein.

Root map

The root map consists of a digital matrix. Each point in the root map has a code which describes the property of that point within the root. An example of this (Fig. C below) shows the coding for a single tier of cells within the meristematic zone. The cytosol is represented by code 0 and the cell wall GPs by codes 1, 2 and 3. The plasma membrane is not represented as a separate entity within the root map but is modelled as part of the cell wall, with different plasma membrane properties (such as polar PIN placement) being included in cell wall properties. We adapted the approach of prescribed PIN protein placement used by Grieneisen *et al*. (2007). PIN protein localisation to the cell wall was modelled by specifying different rate constants (low, medium and high) governing PIN flux from the nearest neighbour cytosolic GP to the cell wall GP, depending on the individual cell wall codes 1, 2 and 3 respectively (kinetic equations and

parameter values are included in Table S1). There are two additional cell wall codes defining PIN activity at the root-shoot cell wall boundary. For the vascular and pericycle boundary cell walls the rate constant for PIN transport is set to zero. Therefore, there is no root to shoot auxin efflux in these cell files. For the epidermal boundary cell walls, the root to shoot auxin efflux is directed by PIN proteins.

```
    E         E         P         V         V         V         V         P         E         E
13333333311333333331111111111111111111111111111111111111111111111111111111111111113333333331133333333 1
1000000002100000000210000000031000000001100000000110000000011000000001300000000120000000012000000001
1000000002100000000210000000031000000001100000000110000000011000000001300000000120000000012000000001
1000000002100000000210000000031000000001100000000110000000011000000001300000000120000000012000000001
1000000002100000000210000000031000000001100000000110000000011000000001300000000120000000012000000001
1000000002100000000210000000031000000001100000000110000000011000000001300000000120000000012000000001
1000000002100000000210000000031000000001100000000110000000011000000001300000000120000000012000000001
1000000002100000000210000000031000000001100000000110000000011000000001300000000120000000012000000001
1000000002100000000210000000031000000001100000000110000000011000000001300000000120000000012000000001
1000000002100000000210000000031000000001100000000110000000011000000001300000000120000000012000000001
1000000002100000000210000000031000000001100000000110000000011000000001300000000120000000012000000001
1000000002100000000210000000031000000001100000000110000000011000000001300000000120000000012000000001
1000000002100000000210000000031000000001100000000110000000011000000001300000000120000000012000000001
1111111111111111111113333333311333333331133333333113333333311333333331133333333111111111111111111111
```

Fig. C Grid point coding for a MZ cell tier in the root map. Cell files: E, epidermal; P, border/pericycle; V, vascular; Grid point codes: 0, cytosol; 1, 2 and 3, cell wall grid points. PIN protein is cycled from the nearest neighbour cytosolic grid point to the cell wall grid points using different rate constants: 1 is low, 2 is medium, 3 is high (kinetic equations and parameter values for localising PIN proteins to cell wall are given in Table S1).

PIN and AUX1 protein localisation to the plasma membrane

The plasma membrane (PM) is not represented as a separate entity within the root map but is modelled as part of the cell wall, with individual plasma membrane properties being included in cell wall properties. Therefore for modelling purposes PIN and AUX1 proteins can be located in a PM/cell wall unified structure. The concentration of PIN protein at a PM/cell wall GP is determined by the cycling and recycling of PIN from the cytosol to and from the PM/cell wall and degradation of PIN within the PM/cell wall. There is no exchange of PIN proteins between PM/cell wall GPs. Cycling of PIN to a PM/cell wall GP is determined by the concentration of PIN at the nearest neighbour cytosolic GP (affected by cytosolic biosynthesis, degradation and flux) and the rate constant for localising PIN proteins to the PM/cell wall GP. The rate constant can be either low, medium or high (kinetic equations and parameter values for localising PIN proteins to cell wall are given in Table S1) and is specified by the cell wall code (1, 2 or 3 respectively) in the root map. The rate of recycling back from the cell wall to the cytosolic GP is determined by PIN concentration at the PM/cell wall, and is inhibited by the cytosolic auxin concentration (Paciorek *et al.*, 2005), and kinetic equations and parameter values for localising PIN proteins to the PM/cell wall are given in Table S1.

AUX1 cycling from the cytosol to and from the PM/cell wall is similar to PIN cycling. It is determined by AUX1 concentrations at the cytosolic and cell wall grid points and two rate constants, one for flux in each direction. These rate constants do not vary between different cell walls. Again, AUX1 degrades within the PM/cell walls as well as in the cytosol and there is no exchange of AUX1 between cell wall GPs.

Flux at the shoot–root boundary

The shoot–root boundary cell wall is located at the proximal end of the root structure. Ethylene and cytokinin can diffuse between the cytosolic GPs of the most proximal cell tier and the boundary cell wall GPs. Auxin shoot to root flux, inhibited by downstream ethylene signalling, is assumed to occur in the pericycle and vascular cell files from the boundary cell wall GPs into the cytosolic GPs, but auxin efflux in the opposite direction is not allowed. Experimental evidence (Suttle, 1988; Chilley *et al*., 2006) indicates that a relatively high ethylene signalling response inhibits the transport of auxin from the shoot to the root tip. However, the molecular basis of this inhibition is unclear. We assume that a molecule or molecules, X, located downstream of ethylene signalling, inhibit the transport of auxin from shoot to root (Liu *et al.*, 2010, 2013). Thus, at the shoot–root boundary cell wall, we assume the auxin flux from shoot to root is described by $\frac{k_{32a}[Auxin]_A}{1+[X]_B/k_{32b}}$ (Liu *et al*., 2010) where $[Auxin]_A$ is the auxin concentration at the cell wall GP of the shoot–root boundary (fixed at 1 μM), and $[X]_B$ is the concentration of ethylene downstream response at the nearest neighbouring cytosolic GP. Thus, auxin shoot to root flux is inhibited by downstream ethylene signalling. We note that, since both k_{32a} and $[Auxin]_A$ are constants, they can be mathematically grouped into one parameter (we have kept them separate in order to maintain the biological clarity). Therefore, the actual auxin concentration at the cell wall GP of the shoot–root boundary, $[Auxin]_A$, is not important on its own for calculating auxin shoot to root flux. In addition, in the model, shoot to root auxin flux is regulated by the concentration of X at the root cytosolic GP that is adjacent to the shoot–root boundary cell wall. Biologically, this implies that the ethylene downstream response at the shoot–root boundary is equal to or similar to the ethylene downstream response at the nearest neighbouring cytosolic GP in the root. This is a reasonable assumption as the two GPs are nearest neighbours.

For the most proximal epidermal cells, PIN-mediated auxin efflux from the cytosolic GPs into the boundary cell wall GPs is allowed but influx is not. The concentrations of ethylene and cytokinin in the boundary cell wall GPs are set such that there is a relatively smooth transition of concentration levels from the root to the shoot. Biologically, this implies that the concentration of ethylene and cytokinin at the shoot–root boundary is equal to or similar to their respective concentrations at the nearest neighbouring cytosolic GP in the root. This is a reasonable assumption as the two GPs are nearest neighbours.

References

Beemster GTS, Baskin IS. 1998. Analysis of cell division and elongation underlying the developmental acceleration of root growth in *Arabidopsis thaliana*. *Plant Physiology* **116**: 1515–1526.

Chilley PM, Casson SA, Tarkowski P, Hawkins N, Wang KL, Hussey PJ, Beale M, Ecker JR, Sandberg GK, Lindsey K. 2006. The POLARIS peptide of Arabidopsis regulates auxin transport and root growth via effects on ethylene signaling. *The Plant Cell* **18**: 3058–3072.

Grieneisen VA, Xu J, Marée AFM, Hogeweg P, Scheres B. 2007. Auxin transport is sufficient to generate a maximum and gradient guiding root growth. *Nature* **449**: 1008–1013.

Liu JL, Mehdi S, Topping J, Tarkowski P, Lindsey K. 2010. Modelling and experimental analysis of hormonal crosstalk in Arabidopsis. *Molecular Systems Biology* **6**: 373.

Liu JL, Mehdi S, Topping J, Friml J, Lindsey K. 2013. Interaction of PLS and PIN and hormonal crosstalk in Arabidopsis root development. *Frontiers in Plant Science* **4**: 75.

Paciorek T, Zazimalova E, Ruthardt N, Petrásek J, Stierhof Y-D, Kleine-Vehn J. Morris DA, Emans N, Jürgens G, Geldner N *et al*. 2005. Auxin inhibits endocytosis and promotes its own efflux from cells. *Nature* **435**: 1251–1256.

Suttle JC. 1988. Effect of ethylene treatment on polar IAA transport, net IAA uptake and specific binding of N-1-naphthylphthalamicacid in tissues and microsomes isolated from etiolated pea epicotyls. *Plant Physiology* **88**: 795–799.

Notes S1 Comparison of modelled auxin concentration trend with experimental DII-VENUS data in the literature

We have compared modelled auxin concentration levels with both IAA2::GUS data and DII-VENUS expression data that we have experimentally measured (see main text for detail). Here we further compare our modelling results with DII-VENUS data in the literature (Brunoud *et al*., 2012; Band *et al*., 2014). DII-VENUS is a fluorescent reporter expressed under the 35S promoter, which is localised to the nucleus and quickly degrades in the presence of auxin. It is considered that DII-VENUS fluorescence more accurately represents relative auxin levels (the inverse of DII-VENUS levels) than other reporters (Brunoud *et al*., 2012; Band *et al*., 2014). The DII-VENUS data in the literature (Brunoud *et al*., 2012; Band *et al*., 2014) were used to compare relative experimental auxin response levels with modelled auxin concentrations for different cell types in the root tip.

The root structure in our model includes epidermal, pericycle, vascular, columella and quiescent centre (QC) cells. Since the root architecture in the literature (Band *et al*., 2014) is more complex than the relatively simple structure in our model, we approximately classify the cell types in our model to match the cell types in the literature (Band *et al*., 2014), as summarised in the following Table.

**Cell type in the literature (Band *et al*., 2014)**	**Corresponding cell type in our model**
Epi M – epidermis, meristem	epidermis, meristem
Epi EZ – epidermis, elongation zone	epidermis, elongation zone
Cor M – cortex, meristem	epidermis, meristem
Cor EZ – cortex, elongation zone	epidermis, elongation zone
End – endodermis	pericycle, meristem
Ste – stele	vascular, meristem
LRC – lateral root cap	not available (NA)
Col – columella	columella
Init – columella initials	not available (NA)
QC – quiescent centre	quiescent centre

To better compare the relative auxin levels for different cell types, we set the auxin level at the QC to be 1, for both experimental data (Band *et al*., 2014) and modelling results. Fig. A below shows the comparison between experimental data (Band *et al*., 2014) and modelling results. The relative auxin levels are plotted in descending order, so that experimental and modelling trends can be easily compared.

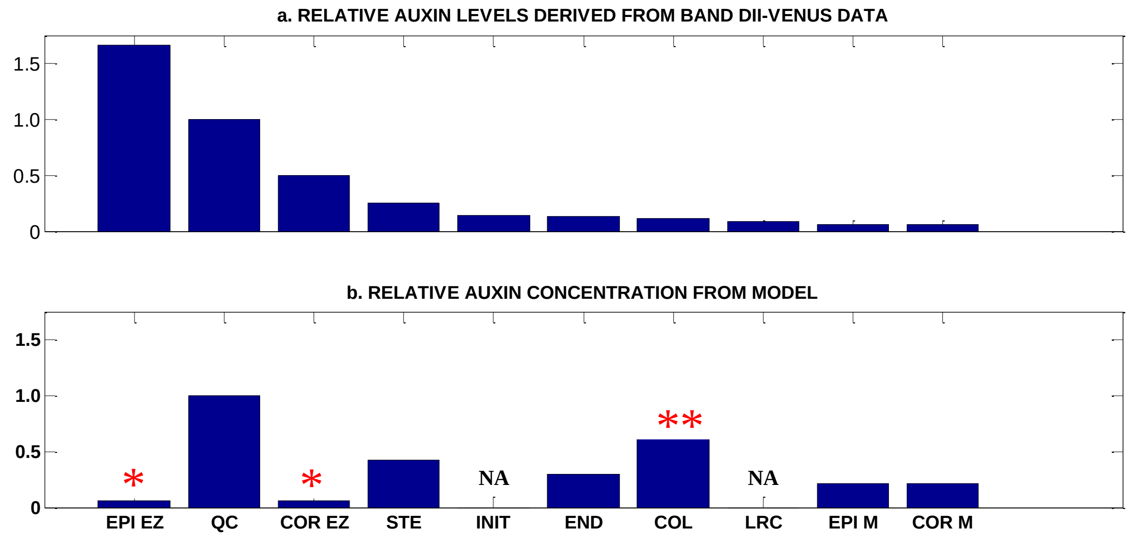

Fig. A Auxin levels (relative to QC). (a) Derived from Fig. 1K (Band *et al*., 2014). (b) Modelling results. * and ** indicate the cell types where the modelled auxin level is below or above the experimental trend (Band *et al*., 2014). EPI, epidermis; COR, cortex; END, endodermis; STE, stele; COL, columella; INIT, columella initials; QC, quiescent centre; M, meristem; EZ, elongation zone; NA, data not available in modelling results.

The trend of the modelled auxin levels for 5 cell types (i.e., quiescent centre, stele, endodermis, epidermis meristem, and cortex meristem) is similar to the trend observed experimentally (Band *et al*., 2014). However, the epidermis and cortex in the elongation zone (marked by *) and the columella (marked by **) are markedly different. These discrepancies between modelling results and experimental observations could be explained as follows.

DII-VENUS expression level measurements rely on the rates of production and decay of DII-VENUS. DII-VENUS expression is under the control of the 35S promoter, while the rate of decay depends both on the levels of auxin and the auxin co-receptors TIR1 and AFB1-5 (Brunoud *et al*., 2012). Homogenous expression of 35S and the auxin co-receptors is therefore necessary to allow representative comparison of relative auxin levels using the DII-VENUS reporter. It was reported that DII-VENUS fluorescence was ubiquitous in *tir1 afb1 afb2 afb3* quadruple mutant roots, which were also significantly less sensitive to auxin (Brunoud *et al*., 2012).The co-receptors TIR1, AFB1 and AFB3 were shown to have very low relative expression (as fusion proteins) in the columella and lateral root cap cells (Fig. S5, Brunoud *et al*., 2012). This could result in the underestimation of relative experimental auxin levels in these cell types and account for the difference between the Band and model results for the columella. Using a non-degradable reporter, mDII-VENUS, the 35S promoter was shown to have significantly increased expression in the transition and elongation zones of the epidermis and cortex (Fig S6A,B in Brunoud *et al.*, 2012), which again could result in underestimation of relative experimental auxin levels in these areas. The DII-VENUS data in the literature (Band *et al*., 2014) suggest that auxin responses in the epidermis and cortex elongation zone are higher than all other regions of the root except the QC. Moreover, by taking into account the effect of increased 35S expression, auxin responses in the epidermis and cortex elongation zone could

possibly exceed those in the QC. This result is not apparent in experimental imaging using the auxin reporter IAA2::GUS (Fig. 3). This discrepancy leads to a number of possibilities. First, the non-degradable reporter, mDII-VENUS, may not fully reflect 35S expression levels in the elongation and transition zones. Second, other unknown factors may suppress DII-VENUS in these zones. Third, there may be additional suppressors of the IAA2::GUS reporter in the transition and elongation zones resulting in variable reporter sensitivity to auxin in different regions of the root. As noted in Band *et al.* (2014), the higher auxin response derived using DII-VENUS data in the elongation zone brings into question the hypothesis that a gradual decrease in auxin levels from the QC maximum determines root developmental zones (Blilou *et al*., 2005; Grieneisen *et al*., 2007).

After taking the above factors into account, our modelling results are in reasonably good agreement with experimental results derived using DII-VENUS data (Band *et al*., 2014).

In addition, we further compare the modelled auxin concentration level with experimental measurement using DII-VENUS for three cell types (Brunoud *et al*., 2012) in the meristem proximal to the QC. Since the root architecture in the literature (Brunoud *et al*., 2012) is more complex than the relatively simple structure in our model, we approximate the classifications of the cell types in our model in terms of the cell types in the literature (Brunoud *et al*., 2012), as summarised in the following table.

**Cell type in the literature (**Brunoud *et al*., 2012**)**	**Corresponding cell type in our model**
stele	vascular, meristem
endodermis and pericycle	pericycle, meristem
cortex and epidermis	epidermis, meristem

Our modelling result (Fig. B below) shows that vascular, pericycle and epidermal cells have a high, medium and low auxin level, respectively. This trend is in agreement with experimental observations (Fig. 2B in Brunoud *et al*., 2012).

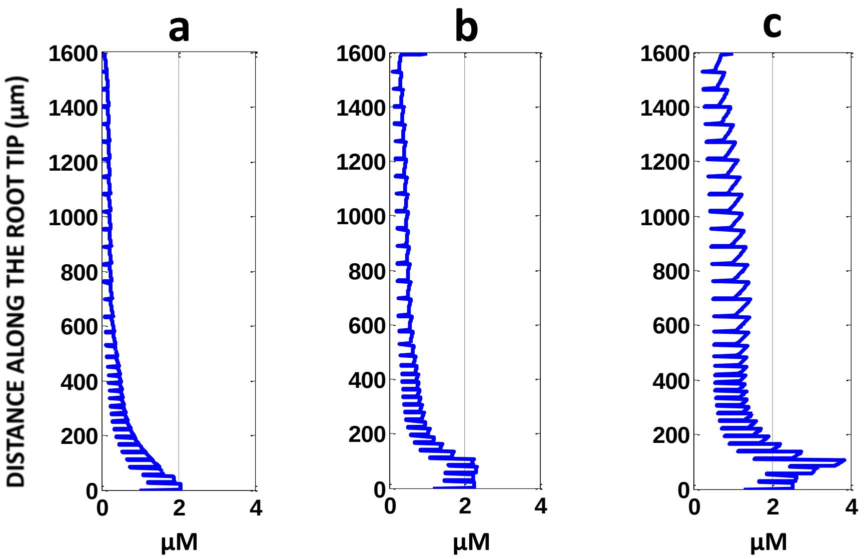

Fig. B Modelled auxin concentration level for three cell types. (a) Epidermal cells; (b) pericycle cells; (c) vascular cells.

In summary, we have compared our modelling results with experimental DII-VENUS data from the literature (Brunoud *et al*., 2012; Band *et al*., 2014). Our modelled auxin concentration trends are in reasonable agreement with experimental observations.

References

Band L. Wells R, Fozard DM, Ghetiu JA, French T, Pound AP, Wilson MP, Yu MH, Li L, Hijazi W. 2014. Systems analysis of auxin transport in the Arabidopsis root apex. *The Plant Cell* **26**: 862–875

Blilou I, Xu J, Wildwater M, WillemsenV, Paponov I, Friml J, Heidstra1 R, Aida M, Palme K, Scheres B. 2005. The PIN auxin efflux facilitator network controls growth and patterning in Arabidopsis roots. *Nature* **433**: 39–44.

Brunoud G, Wells DM, Oliva M, Larrieu A, Mirabet V, Burrow AH, Beeckman T, Kepinski S, Traas J, Bennett MJ *et al*. 2012. A novel sensor to map auxin response and distribution at high spatio-temporal resolution. *Nature* **482**: 103–106.

Grieneisen VA, Xu J, Marée AFM, Hogeweg P, Scheres B. 2007. Auxin transport is sufficient to generate a maximum and gradient guiding root growth. *Nature* **449**: 1008–1013.

Notes S2 Evaluation of model sensitivity

While the modelling equations must be formulated in specific forms for describing the kinetics of all processes as described in Fig. 2, many parameter sets can be fitted against experimental data as the number of parameters is much more than that of experimental observations. By examining parameters randomly, we find that, when a parameter changes, if we allow at least one or more other parameters to change, we can find a new set of parameters that meet the criteria for model fitting (see section '**Model fitting reveals that both PIN and AUX1 activities must be restricted to certain ranges in order to generate correct auxin patterning'**). The model using the new set of parameters also makes correct predictions, leading to the conclusions drawn in this work. For example, we have systematically examined all results in this paper for three randomly-generated sets of parameters that meet the criteria for defining a wildtype, and find that all modelling results for the three sets of parameters are qualitatively similar. The set of parameters we have used to generate all results in this work is included in Table S1.

We have subsequently evaluated model sensitivity to changes in the parameter values defining the wildtype root (Table S1). The parameters used for sensitivity analysis include diffusion rates, auxin decay rate, cytokinin biosynthesis rate and *AUX1* and *PIN* transcription rates. The valuation of model sensitivity has shown that the model is robust to variations in the parameter values, as detailed below.

Changes to the auxin diffusion constant have little effect on average auxin concentrations and auxin patterning in the root. If the auxin diffusion constant is very small ($< 20\ \mu m^2\ s^{-1}$), the auxin transport rate is reduced and auxin patterning no longer emerges. Changes to the cytokinin and ethylene diffusion constants have an even smaller impact on auxin concentrations and patterning than changes to the auxin diffusion constant. The PIN protein diffusion constant affects the ratio of PIN proteins in the plasma membrane to that in the cytosol. At very low PIN diffusion constant values ($<20\ \mu m^2\ s^{-1}$), PIN proteins predominantly localise to the cytosol, limiting auxin transport such that no auxin maximum emerges. As the PIN diffusion constant increases, PIN at the plasma membrane increases, which in turn increases the rate of auxin efflux and an auxin maximum emerges. Auxin patterning can emerge for a wide range of PIN diffusion constants ($>=20\ \mu m^2\ s^{-1}$ and $<= 1000\ \mu m^2\ s^{-1}$). Changing the AUX1 diffusion constant has similar effects and the formation of the auxin maximum is very robust to changes in the AUX1 diffusion rate ($>=5\ \mu m^2\ s^{-1}$ and $<=1500\ \mu m^2\ s^{-1}$).

The coordinated rates of expression of PIN and AUX1 proteins control auxin transport through the root. The relative expression level of the two transporter proteins determines the proportion of auxin in the cytosol to the cell walls, with high relative PIN expression driving auxin into the cell walls and high relative AUX1 expression concentrating auxin in the cytosol. The importance of the coordinated permeability of the two transporter proteins is also demonstrated by simultaneously increasing PIN and AUX1 protein expression levels.

Due to the discrepancy between modelling results and experimental observations for cytokinin patterning, it is important to evaluate the model sensitivity to the parameter changes relating to cytokinin. Auxin patterning proved to be very robust to changes in all parameters relating to cytokinin diffusion, biosynthesis and degradation.

Model sensitivity analysis also shows that auxin patterning always emerges if the auxin decay rate constant is reduced to 50% or increased to 200% of the reference wildtype value.

In summary, model sensitivity analysis shows that the modelling results are robust to variation in parameter values and that there is a wide range of values that meet the criteria for generating correct auxin patterning.
